# Synthesis and Biological Evaluation of Carbocyclic Analogues of Pachastrissamine

**DOI:** 10.3390/md13020824

**Published:** 2015-02-03

**Authors:** Yongseok Kwon, Jayoung Song, Hoon Bae, Woo-Jung Kim, Joo-Youn Lee, Geun-Hee Han, Sang Kook Lee, Sanghee Kim

**Affiliations:** College of Pharmacy, Seoul National University, Seoul 151-742, Korea; E-Mails: chemys@snu.ac.kr (Y.K.); poppy27@snu.ac.kr (J.S.); hoonbae1234@gmail.com (H.B.); lwjkim@snu.ac.kr (W.-J.K.); leejooyoun@snu.ac.kr (J.-Y.L.); thariel@snu.ac.kr (G.-H.H.); sklee61@snu.ac.kr (S.K.L.)

**Keywords:** pachastrissamine, jaspine B, carbocyclic analogue, cytotoxicity, sphingosine kinase inhibitor, molecular modeling

## Abstract

A series of carbocyclic analogues of naturally-occurring marine sphingolipid pachastrissamine were prepared and biologically evaluated. The analogues were efficiently synthesized via a tandem enyne/diene-ene metathesis reaction as a key step. We found that the analogue **4b** exhibited comparable cytotoxicity and more potent inhibitory activity against sphingosine kinases, compared to pachastrissamine. Molecular modeling studies were conducted to provide more detailed insight into the binding mode of **4b** in sphingosine kinase. In our docking model, pachastrissamine and **4b** were able to effectively bind to the binding pocket of sphingosine kinase 1 as co-crystalized sphingosine. However, **4b** showed a hydrophobic interaction with Phe192, which suggests that it contributes to its increased inhibitory activity against sphingosine kinase 1.

## 1. Introduction

**Figure 1:**
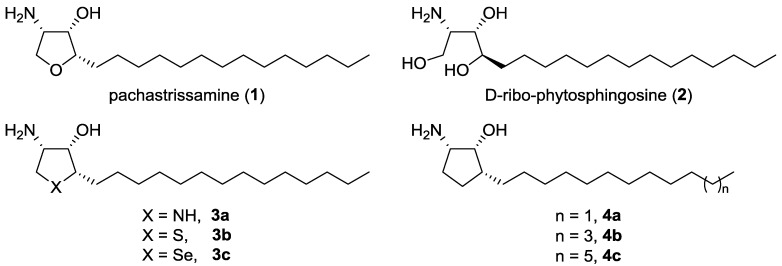
Chemical structures of Compounds **1**–**4**.

Pachastrissamine (jaspine B, **1**; Figure 1) is a natural anhydrous derivative of phytosphingosine **2**, possessing a tetrahydrofuran core with three contiguous stereogenic centers. It was first isolated by Higa *et al.* from marine sponge *Pachastrissa* sp. in 2002 [1] and subsequently isolated from the Vanuatuan marine sponge *Jaspis* sp. by Debitus *et al.* in 2003 [2]. This marine natural product showed significant cytotoxicity against various cancer cell lines, such as P388, MEL28, A549, HT29 and HeLa [1,3]. It was found to have an inhibitory activity against sphingomyelin synthase, which, in turn, triggers apoptosis in tumor cells via a caspase-dependent pathway [4]. It was also reported that pachastrissamine and its stereoisomers inhibit sphingosine kinases (SphKs) and atypical protein kinase C [5]. Because of its intriguing biological activity, it has been an interesting target for synthetic chemists, and various synthetic routes to pachastrissamine have been reported [6,7,8,9,10,11,12,13,14,15,16]. However, the structure-activity relationship (SAR) of pachastrissamine remains relatively unreported. Génisson *et al.* described analogues with a modified aliphatic chain, which exhibited cytotoxicity comparable to or lower than pachastrissamine on two distinct cancer cell lines (B16 and A375 melanoma cell lines) [17,18]. Delgado *et al.* reported the stereoisomers of pachastrissamine as being 10- to 20-times less potent than the natural one [19]. Recently, Liu *et al.* reported pachastrissamine analogues containing a 1,2,3-triazole ring in the alkyl chain [20]. One analogue showed better increased cytotoxicity than the natural compound. These results suggest that the configuration and aliphatic chain of pachastrissamine would be essential to retain biological activity. The contribution of the tetrahydrofuran ring to its biological profile has been explored. The aza-analogues of pachastrissamine **3a** were reported by Génisson *et al.* [21], and the sulfur and selenium analogues **3b**–**c** were previously reported by our group [22]. They exhibited comparable potency to natural pachastrissamine, indicating that the ring oxygen atom of pachastrissamine could be replaced with bioisosteres.

In this regard, we designed carbocyclic analogues, replacing the ethereal oxygen (–O–) with a methylene group (–CH_2_–) as a divalent bioisostere [23]. The advantages of carbocyclic analogues of natural product have already been demonstrated by several successful examples, such as carbasugars and carbocyclic nucleosides [24,25]. As seen in these successful precedents, we expected that the carbocyclic analogue would show enhanced chemical and metabolic stability compared to the parent natural product. In addition, from this study, we would be able to decipher the SAR involved in the role of ethereal oxygen, which is valuable information in the development of new anti-cancer therapeutic agents. Herein, we report the synthesis and biological evaluation of a series of carbocyclic pachastrissamine analogues **4** (Figure 1) in which the alkyl-chain lengths have been varied.

## 2. Results and Discussion

### 2.1. Chemistry

#### 2.1.1. Retrosynthetic Analysis

**Scheme 1:**
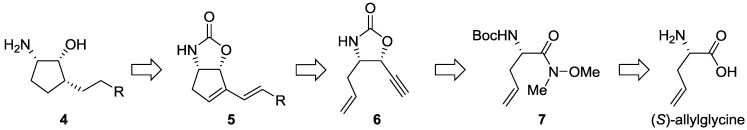
Retrosynthetic plan.

Our retrosynthetic plan for the synthesis of **4** is shown in Scheme 1. The designed analogues **4** would be accessed from diene **5** using catalytic hydrogenation and hydrolysis. We expected catalytic hydrogenation of **5** would occur from the convex face of the bicyclic system to afford the desired stereochemistry [6]. The requisite diene **5** would be derived from the ring-closing metathesis (RCM) of enyne **6** and subsequent cross-metathesis (CM) between the resulting diene and the appropriate olefin in a tandem fashion [26,27,28,29]. Substrate **6** would be derived from a known amide **7**, which, in turn, can be easily prepared from commercially available (*S*)-allylglycine.

#### 2.1.2. Synthesis

**Scheme 2:**
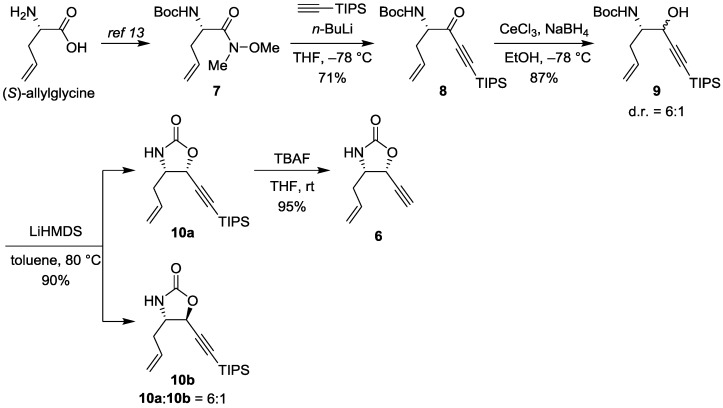
Synthesis of enyne **6**. TIPS: triisopropylsilyl; LiHMDS: lithium hexamethyldisilazide; TBAF: tetrabutylammonium fluoride.

Our synthesis, as shown in Scheme 2, commenced with the preparation of a known Weinreb amide **7** from commercially available (*S*)-allylglycine in two steps using a previously reported procedure [30]. Treatment of amide **7** with lithium acetylide in THF yielded ynone **8** in a 71% yield. The stereoselective reduction of ynone **8** was explored with various hydride reagents. After several trials, it was found that the treatment of **8** with sodium borohydride and cerium chloride at low temperature gave an inseparable diastereomeric mixture of alcohol **9** with the best selectivity (diastereomeric ratio = 6:1) in 87% yield. The obtained alcohol **9** was treated with lithium hexamethyldisilazide in hot toluene to provide oxazolidinone **10** in a high yield (90%). At this stage, both diastereomers (**10a** and **10b**) were separated via column chromatography, and the relative configuration of the diastereomers was assigned by a 2D-NOESY experiment (Figures S7 and S10 in the Supplementary Materials). The triisopropylsilyl group of major diastereomer **10a** was easily removed with tetrabutylammonium fluoride to give the terminal acetylene **6** in a 95% yield.

**Scheme 3:**
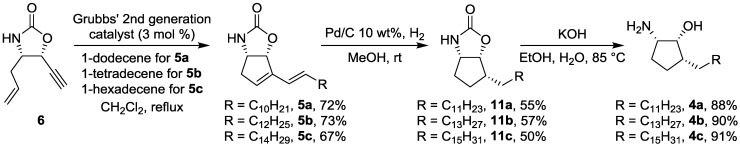
Synthesis of carbocyclic analogues, **4a**–**c**.

With the terminal enyne **6** in hand, the tandem enyne/diene-ene metathesis reaction was carried out (Scheme 3). The reaction of **6** with the appropriate alkenes in the presence of second-generation Grubbs catalyst (3 mol%) afforded the desired diene **5a**–**c** with exclusive (*E*)-geometry. The catalytic hydrogenation of diene **5a**–**c** was accomplished to give cyclopentane **11a**–**c** as the only stereoisomers detected by ^1^H NMR. Finally, hydrolysis of the oxazolidinone with KOH in refluxing EtOH afforded the desired carbocyclic analogues, **4a**–**c**.

### 2.2. Biological Evaluation

#### 2.2.1. Cell Viability

The cytotoxic activity of analogues **4a**–**c** was examined in various cancer cell lines (Table 1) by the sulforhodamine B (SRB) assay [31]. For a direct comparison, natural pachastrissamine (**1**) was employed as a positive control. It was found that the cytotoxic activities of carbocyclic analogues **4** were influenced by the length of the alkyl chain. The analogue, **4b**, which has the same chain length as pachastrissamine, exhibited a comparable potency to the parent natural product, **1**, against various cell lines. However, both the shorter and longer chain analogues, **4a** and **4c**, were less effective than pachastrissamine.

**Table 1:** Cytotoxic activity of pachastrissamine (**1**) and its carbocyclic analogues, **4a**–**c**.

Compound	IC_50_ (μM) ^a^				
	HCT-116 ^b^	SNU-638 ^c^	MDA-MB-231 ^d^	PC-3 ^e^	Caki-1 ^f^
**1**	1.0	1.2	0.7	0.7	1.7
**4a**	9.7	12.8	6.1	7.6	12.1
**4b**	1.0	1.7	1.8	1.0	3.2
**4c**	3.2	4.4	4.1	3.1	6.6

^a^ 50% inhibition concentration; ^b^ HCT-116, human colon cancer cell line; ^c^ SNU-638, human stomach cancer cell line; ^d^ MDA-MB-231, human breast cancer cell line; ^e^ PC-3, human prostate cancer cell line; ^f^ Caki-1, human renal cancer cell line.

#### 2.2.2. Inhibitory Activity against Sphingosine Kinases

Because pachastrissamine was reported as an inhibitor of sphingosine kinases (SphKs), the inhibitory activity of analogues **4** against SphKs were examined using a sphingosine kinase inhibition assay (Table 2). There are two isoforms of mammalian SphKs (SphK1 and SphK2) catalyzing the phosphorylation of sphingosine to sphingosine 1-phosphate (S1P). S1P regulates diverse cellular processes, including cell growth, survival and differentiation [32]. In our experiments, *N*,*N*-dimethylsphingosine (DMS), as well as pachastrissamine (**1**) were used as a positive control [33]. It was revealed that the inhibitory activity of **4** against SphKs was influenced by the length of the attached chain. The analogue **4b** showed a similar inhibitory activity to DMS, which, in turn, was more effective than **1**. However, the other analogues, **4a** and **4c**, were found to be less effective at the inhibition of SphKs. These results suggest that the ring oxygen atom of pachastrissamine is dispensable, but the appropriate chain length is apparently required to retain its biological properties.

**Table 2:** Inhibitory activity of pachastrissamine (**1**) and its carbocyclic analogues, **4a**–**c**, against sphingosine kinases.

Compound	IC_50_ (μM) ^a^	
	SphK1	SphK2
**1**	12.0	41.8
**4a**	58.2	>100
**4b**	7.5	20.1
**4c**	41.3	>100
DMS ^b^	6.6	19.9

^a^ 50% inhibition concentration; ^b^ *N*,*N*-dimethylsphingosine.

### 2.3. Molecular Modeling

**Figure 2:**
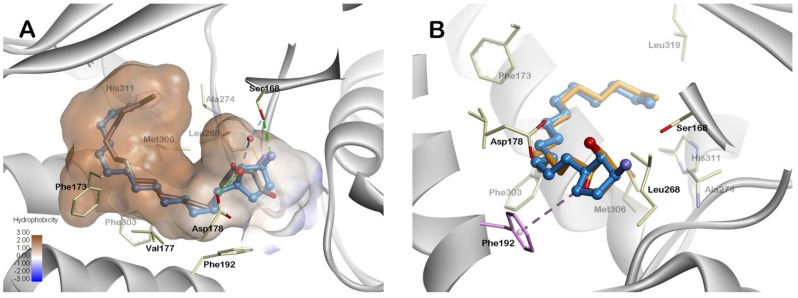
Docking model of the carbocyclic analogue **4b** and pachastrissamine (**1**). (**A**) The overlay of the proposed binding model of carbocyclic analogue **4b** (blue) and X-ray complex sphingosine (gray). A water molecule is shown as a red sphere. The direct H-bonds and water-mediated H-bonds are shown as green and blue dashed lines, respectively. The ligand binding surface of the binding pocket was colored according to hydrophobicity (brown: hydrophobic, blue: hydrophilic); (**B**) The overlay of the proposed binding model of **4b** (blue) and **1** (orange). Hydrophobic interaction with Phe192 is shown as a pink dashed line.

To understand the molecular interaction between **4b** and the sphingosine kinase, a molecular modeling study was performed using the crystal structure of human SphK1 in complex with sphingosine (PDB Code 3VZB) [34]. As shown in Figure 2, pachastrissamine (**1**) and its carbocyclic analogue **4b** are able to effectively bind to the binding pocket of SphK1 in virtually the same pose. They occupy nearly the same position as sphingosine. In our docking model, the alkyl chain of **4b** is buried in the hydrophobic J-shaped tunnel constituted of hydrophobic residues Leu268, Ala274, His311, Phe173, Phe303, Val177 and Phe192. The hydroxyl group forms a hydrogen bond with Asp178 and a water-mediated hydrogen bond with Ser168, in the same manner as the 3-hydroxyl group of sphingosine. The amino group makes a hydrogen bond interaction with Ser168. In the hydrophilic recognition site, pachastrissamine (**1**) and its analogue **4b** formed the same set of hydrogen bonds. However, carbocyclic analogue **4b** exhibits an additional interaction with the hydrophobic residue, Phe192 (Figure 2B). This pi-alkyl interaction seems to contribute to the increased activity of **4b** toward SphK1 compared to **1** [35].

## 3. Experimental Section

### 3.1. Chemistry

#### 3.1.1. General

All chemicals were reagent grade and used as purchased. All reactions were performed under an inert atmosphere of dry nitrogen using distilled dry solvents. Reactions were monitored through thin layer chromatography (TLC) analysis using silica gel 60 F-254 thin layer plates. Compounds were visualized on the TLC plates under UV light and by spraying with either KMnO_4_ or anisaldehyde staining solutions. Flash column chromatography was conducted on silica gel 60 (230–400 mesh). Melting points were measured using a Buchi B-540 melting point apparatus without correction. ^1^H NMR (400, 500 or 600 MHz) and ^13^C NMR (100, 125 or 150 MHz) spectra were recorded in δ units relative to the deuterated solvent. The IR spectra were measured on a Fourier transform infrared spectrometer. High-resolution mass spectra (HRMS) were recorded using FAB.

#### 3.1.2. *tert*-Butyl (*S*)-(5-oxo-7-(triisopropylsilyl)hept-1-en-6-yn-4-yl)carbamate (**8**)

To a solution of (triisopropylsilyl)acetylene (17.4 mL, 77.4 mmol) in THF (80 mL) was added *n*-BuLi (48.4 mL, 77.4 mmol, 1.6 M solution in hexane) at −78 °C under nitrogen. After the reaction mixture was stirred for 30 min at −78 °C, known amide **7** (10 g, 38.7 mmol) in dry THF (20 mL) was added at 0 °C. The reaction mixture was stirred for 1.5 h at 0 °C. The reaction was quenched with a saturated NH_4_Cl aqueous solution and extracted with EtOAc. The organic layer was washed twice with brine, dried over MgSO_4_, filtered and concentrated. The crude product was purified using column chromatography on silica gel (hexane/EtOAc, 20:1), to afford the desired ynone **8** (10.4 g, 71%) as a yellow oil. ${\left[\text{α}\right]}_{\text{D}}^{20}$ +8.20 (c 1.0, CHCl_3_); ^1^H NMR (400 MHz, CDCl_3_) δ 5.70–5.61 (m, 1H), 5.13 (d, *J* = 17.2 Hz, 1H), 5.12 (d, *J* = 9.2 Hz, 1H), 5.10 (brs, 1H), 4.48 (d, *J* = 5.7 Hz, 1H), 2.70–2.59 (m, 2H), 1.42 (s, 9H), 1.17–1.01 (m, 21H); ^13^C NMR (100 MHz, CDCl_3_) δ 185.4, 155.1, 131.7, 119.5, 102.2, 100.1, 80.0, 60.8, 36.0, 28.3 (3C), 18.5 (6C), 11.0 (3C); IR (CHCl_3_) ν_max_ 3358, 2947, 2869, 2150, 1719, 1682, 1495, 1368, 1171 (cm^−1^); HRMS (FAB) calcd. for C_21_H_38_NO_3_Si ([M + H]^+^), 380.2621; found, 380.2614.

#### 3.1.3. *tert*-Butyl ((4*S*)-5-hydroxy-7-(triisopropylsilyl)hept-1-en-6-yn-4-yl)carbamate (**9**)

To a solution of ynone **8** (10 g, 26.3 mmol) in EtOH (100 mL) was added anhydrous CeCl_3_ (13 g, 52.6 mmol) at room temperature under nitrogen. After the reaction mixture was stirred for 15 min, NaBH_4_ (2.0 g, 52.6 mmol) was added at −78 °C. The reaction mixture was stirred for 3 h at −78 °C. The reaction was quenched with a saturated NH_4_Cl aqueous solution and extracted with EtOAc. The organic layer was washed twice with brine, dried over MgSO_4_, filtered and concentrated. The crude product was purified using column chromatography on silica gel (hexane/EtOAc, 5:1), to afford the desired alcohol **9** (8.7 g, 87%) as a diastereomeric mixture (6:1) as a colorless oil. ${\left[\text{α}\right]}_{\text{D}}^{20}$ −18.3 (c 1.0, CHCl_3_); ^1^H NMR (400 MHz, CDCl_3_) δ 5.82–5.72 (m, 1H), 5.08 (d, *J* = 15.2 Hz, 1H), 5.06 (d, *J* = 8.9 Hz, 1H), 4.74–4.72 (m, 0.85H), 4.45–4.44 (m, 1H), 3.82 (brs, 0.85H), 3.72 (brs, 0.15H), 3.38 (brs, 0.85H), 2.56–2.46 (m, 0.15H), 2.42–2.22 (m, 1.85H), 1.40 (s, 9H), 1.04 (s, 21H); ^13^C NMR (100 MHz, CDCl_3_) δ 156.5, 134.0, 118.0, 105.2, 87.6, 79.9, 66.1, 55.1, 35.7, 28.2 (3C), 18.5 (6C), 11.1 (3C); IR (CHCl_3_) ν_max_ 3428, 2945, 2868, 2170, 1696, 1504, 1170 (cm^−1^); HRMS (FAB) calcd. for C_21_H_40_NO_3_Si ([M + H]^+^), 382.2777; found, 382.2770.

#### 3.1.4. (4*S*)-4-Allyl-5-((triisopropylsilyl)ethynyl)oxazolidin-2-one (**10**)

To a solution of alcohol **9** (8 g, 21 mmol) in toluene (100 mL) was added lithium hexamethyldisilazide (46.2 mL, 46.2 mmol, 1 M solution in THF) at room temperature under nitrogen. After stirring for 1.5 h at 80 °C, the reaction mixture was cooled to 0 °C. The reaction was quenched with a saturated NH_4_Cl aqueous solution and extracted with EtOAc. The organic layer was washed twice with brine, dried over MgSO_4_, filtered and concentrated. The crude product was purified using column chromatography on silica gel (hexane/EtOAc, 1.5:1), to afford the desired oxazolidinone **10a** (5.0 g, 77%) and **10b** (850 mg, 13%).

(4*S*,5*R*)-4-Allyl-5-((triisopropylsilyl)ethynyl)oxazolidin-2-one (**10a**): a white solid; m.p. 91.2–93.3 °C; ${\left[\text{α}\right]}_{\text{D}}^{20}$ −4.12 (c 0.35, CHCl_3_); ^1^H NMR (400 MHz, CDCl_3_) δ 5.80–5.72 (m, 1H), 5.33–5.31 (d, *J* = 7.8 Hz, 1H), 5.20 (d, *J* = 10.4 Hz, 1H), 5.19 (d, *J* = 16.8 Hz, 1H), 5.17 (brs, 1H), 3.88 (td, *J* = 4.1 Hz, 8.6 Hz, 1H), 2.59–2.53 (m, 1H), 2.44–2.36 (m, 1H), 1.07 (s, 21H); ^13^C NMR (100 MHz, CDCl_3_) δ 159.6, 132.7, 119.8, 98.5, 93.5, 70.1, 54.1, 36.8, 18.5 (6C), 11.0 (3C); IR (CHCl_3_) ν_max_ 3276, 2946, 2868, 1759, 1464, 1334, 1048, 758, 676 (cm^−1^); HRMS (FAB) calcd. for C_17_H_30_NO_2_Si ([M + H]^+^), 308.2046; found, 308.2039.

(4*S*,5*S*)-4-Allyl-5-((triisopropylsilyl)ethynyl)oxazolidin-2-one (**10b**): a yellow oil; ${\left[\text{α}\right]}_{\text{D}}^{20}$ −36.2 (c 1.4, CHCl_3_); ^1^H NMR (600 MHz, CDCl_3_) δ 6.12 (d, *J* = 9.2 Hz, 1H), 5.77–5.70 (m, 1H), 5.19 (d, *J* = 11.5 Hz, 1H), 5.18 (d, *J* = 16.1 Hz, 1H), 4.80 (d, *J* = 6.4 Hz, 1H), 3.87 (q, *J* = 6.4 Hz, 1H), 2.40 (q, *J* = 6.7 Hz, 1H), 2.33 (q, *J* = 7.1 Hz, 1H), 1.05 (s, 21H); ^13^C NMR (150 MHz, CDCl_3_) δ 158.2, 131.5, 119.8, 101.7, 90.8, 70.9, 59.2, 38.7, 18.5 (6C), 11.0 (3C); IR (CHCl_3_) ν_max_ 3282, 2946, 2869, 1760, 1464, 1323, 1071, 992, 883, 675 (cm^−1^); HRMS (FAB) calcd. for C_17_H_30_NO_2_Si ([M + H]^+^), 308.2046; found, 308.2042.

#### 3.1.5. (4*S*,5*R*)-4-Allyl-5-ethynyloxazolidin-2-one (**6**)

To a solution of alcohol **10a** (4.0 g, 13.0 mmol) in THF (60 mL) was added tetrabutylammonium fluoride (15.6 mL, 15.6 mmol, 1 M solution in THF) at room temperature under nitrogen. The reaction mixture was stirred for 1 h at room temperature. The reaction was quenched with a saturated NH_4_Cl aqueous solution and extracted with EtOAc. The organic layer was washed twice with brine, dried over MgSO_4_, filtered and concentrated. The crude product was purified using column chromatography on silica gel (hexane/EtOAc, 1:1), to afford the desired terminal acetylene **6** (1.87 g, 95%) as a white solid. m.p. 42.0–44.0 °C; ${\left[\text{α}\right]}_{\text{D}}^{20}$ −6.32 (c 0.54, CHCl_3_); ^1^H NMR (400 MHz, CDCl_3_) δ 5.81–5.70 (m, 1H), 5.28 (dd, *J* = 2.2 Hz, 7.8 Hz, 2H), 5.22 (d, *J* = 0.7 Hz, 1H), 5.19–5.18 (m, 1H), 3.91 (td, *J* = 4.1 Hz, 8.6 Hz, 1H) 2.72 (d, *J* = 2.1 Hz, 1H), 2.58–2.52 (m, 1H), 2.44–2.36 (m, 1H); ^13^C NMR (150 MHz, CDCl_3_) δ 157.2, 132.4, 120.0, 79.0, 75.8, 69.3, 53.9, 36.5; IR (CHCl_3_) ν_max_ 3292, 2128, 1755, 1644, 1338, 1243, 1041 (cm^−1^); HRMS (FAB) calcd. for C_8_H_10_NO_2_ ([M + H]^+^), 152.0712; found, 152.0714.

#### 3.1.6. General Procedure for the Preparation of **5**

To a solution of **6** (300 mg, 2.0 mmol) with the appropriate alkene (2 mL) in CH_2_Cl_2_ (20 mL) was added Grubbs’ second-generation catalyst (50 mg, 0.059 mmol) at room temperature. The resulting mixture was refluxed for 20 h. After the mixture was cooled to room temperature, the solvent was removed under reduced pressure. The crude product was purified by column chromatography on silica gel (hexane/EtOAc, 1:3) to give diene **5**.

#### 3.1.7. (3a*S*,6a*R*)-6-((*E*)-Dodec-1-en-1-yl)-3,3a,4,6a-tetrahydro-2*H*-cyclopenta[*d*]oxazol-2-one (**5a**)

A white solid (420 mg, 72%); m.p. 83.6–84.5 °C; ${\left[\text{α}\right]}_{\text{D}}^{20}$ +23.7 (c 1.1, CHCl_3_); ^1^H NMR (500 MHz, CDCl_3_) δ 6.12 (d, *J* = 16.0 Hz, 1H), 5.94 (td, *J* = 7.4 Hz, 15.2 Hz, 1H), 5.68 (s, 1H), 5.63 (brs, 1H), 5.59 (d, *J* = 7.8 Hz, 1H), 4.44 (t, *J* = 7.2 Hz, 1H), 2.71 (dd, *J* = 6.1 Hz, 18.1 Hz, 1H), 2.47 (d, *J* = 18.2 Hz, 1H), 2.15–2.02 (m, 2H), 1.40–1.35 (m, 2H), 1.24 (s, 14H), 0.86 (t, *J* = 6.9 Hz, 3H); ^13^C NMR (125 MHz, CDCl_3_) δ 158.9, 139.8, 135.0, 128.8, 123.0, 84.9, 53.7, 40.2, 33.1, 31.9, 29.62, 29.58, 29.48, 29.33, 29.25, 29.0, 22.7, 14.1; IR (CHCl_3_) ν_max_ 3270, 2922, 2854, 1739, 1716, 1385, 1238, 1018, 756 (cm^−1^); HRMS (FAB) calcd. for C_18_H_30_NO_2_ ([M + H]^+^), 292.2277; found, 292.2279.

#### 3.1.8. (3a*S*,6a*R*)-6-((*E*)-Tetradec-1-en-1-yl)-3,3a,4,6a-tetrahydro-2*H*-cyclopenta[*d*]oxazol-2-one (**5b**)

A white solid (466 mg, 73%); m.p. 93.1–94.5 °C; ${\left[\text{α}\right]}_{\text{D}}^{20}$ +33.0 (c 0.43, CHCl_3_); ^1^H NMR (400 MHz, CDCl_3_) δ 6.12 (d, *J* = 15.9 Hz, 1H), 5.94 (td, *J* = 7.3 Hz, 15.1 Hz, 1H), 5.68 (s, 1H), 5.59 (d, *J* = 7.7 Hz, 1H), 5.51 (brs, 1H), 4.44 (t, *J* = 7.1 Hz, 1H), 2.72 (dd, *J* = 6.0 Hz, 18.3 Hz, 1H), 2.47 (d, *J* = 18.2 Hz, 1H), 2.13–2.04 (m, 2H), 1.40–1.37 (m, 2H), 1.24 (s, 18H), 0.86 (t, *J* = 6.7 Hz, 3H); ^13^C NMR (100 MHz, CDCl_3_) δ 158.8, 139.8, 135.1, 128.7, 123.0, 84.9, 53.7, 40.2, 33.1, 31.9, 29.7 (2C), 29.64, 29.58, 29.48, 29.35, 29.3, 29.0, 22.7, 14.1; IR (CHCl_3_) ν_max_ 3244, 2917, 2852, 1735, 1471, 1386, 1248, 1016, 716 (cm^−1^); HRMS (FAB) calcd. for C_20_H_34_NO_2_ ([M + H]^+^), 320.2590; found, 320.2584.

#### 3.1.9. (3a*S*,6a*R*)-6-((*E*)-Hexadec-1-en-1-yl)-3,3a,4,6a-tetrahydro-2*H*-cyclopenta[*d*]oxazol-2-one (**5c**)

A white solid (466 mg, 67%); m.p. 90.1–91.6 °C; ${\left[\text{α}\right]}_{\text{D}}^{20}$ +38.4 (c 0.95, CHCl_3_); ^1^H NMR (600 MHz, CDCl_3_) δ 6.12 (d, *J* = 16.0 Hz, 1H), 5.94 (td, *J* = 7.4 Hz, 15.3 Hz, 1H), 5.86 (brs, 1H), 5.68 (s, 1H), 5.59 (d, *J* = 8.2 Hz, 1H), 4.44 (t, *J* = 7.1 Hz, 1H), 2.70 (dd, *J* = 6.4 Hz, 17.9 Hz, 1H), 2.47 (d, *J* = 18.3 Hz, 1H), 2.15–2.02 (m, 2H), 1.39–1.34 (m, 2H), 1.24 (s, 22H), 0.86 (t, *J* = 7.1 Hz, 3H); ^13^C NMR (150 MHz, CDCl_3_) δ 159.0, 139.8, 135.0, 128.9, 123.0, 84.9, 53.8, 40.2, 33.1, 31.9, 29.67 (3C), 29.64 (2C), 29.58, 29.48, 29.35, 29.26, 29.0, 22.7, 14.1; IR (CHCl_3_) ν_max_ 3284, 2922, 2853, 1737, 1716, 1385, 1237, 1015 (cm^−1^); HRMS (FAB) calcd. for C_22_H_38_NO_2_ ([M + H]^+^), 348.2903; found, 348.2905.

#### 3.1.10. General Procedure for the Preparation of **11**

To a solution of diene **5** (0.72 mmol) in MeOH (30 mL) was added Pd/C (40 mg, 10 wt.% Pd) at room temperature. The reaction mixture was shaken in a Parr hydrogenator for 10 h at an initial pressure of 35 psi. The catalyst was filtered off with Celite^®^ (OCI Company Ltd., Seoul, Korea), and the solvent was removed under reduced pressure. The crude product was purified by column chromatography on silica gel (hexane/EtOAc, 1:3) to give the desired product, **11**.

#### 3.1.11. (3a*S*,6*R*,6a*R*)-6-Dodecylhexahydro-2*H*-cyclopenta[*d*]oxazol-2-one (**11a**)

A white solid (117 mg, 55%). m.p. 67.8–69.1 °C; ${\left[\text{α}\right]}_{\text{D}}^{20}$ +10.6 (c 0.50, CHCl_3_); ^1^H NMR (400 MHz, CDCl_3_) δ 5.71 (brs, 1H), 4.88 (t, *J* = 6.1 Hz, 1H), 4.22 (t, *J* = 6.5 Hz, 1H), 1.76–1.69 (m, 3H), 1.63–1.55 (m, 2H), 1.53–1.39 (m, 2H), 1.23 (s, 20H), 0.86 (t, *J* = 6.7 Hz, 3H); ^13^C NMR (100 MHz, CDCl_3_) δ 160.1, 83.2, 56.6, 46.0, 33.8, 31.9, 29.8, 29.62 (4C), 29.56, 29.3, 28.4, 28.2, 28.0, 22.7, 14.1; IR (CHCl_3_) ν_max_ 3306, 2923, 2854, 1736, 1713, 1423, 1242 (cm^−1^); HRMS (FAB) calcd. for C_18_H_34_NO_2_ ([M + H]^+^), 296.2590; found, 296.2588.

#### 3.1.12. (3a*S*,6*R*,6a*R*)-6-Tetradecylhexahydro-2*H*-cyclopenta[*d*]oxazol-2-one (**11b**)

A white solid (127 mg, 57%). m.p. 88.7–89.6 °C; ${\left[\text{α}\right]}_{\text{D}}^{20}$ +8.56 (c 0.52, CHCl_3_); ^1^H NMR (400 MHz, CDCl_3_) δ 5.59 (brs, 1H), 4.88 (t, *J* = 6.1 Hz, 1H), 4.23 (t, *J* = 6.5 Hz, 1H), 1.77–1.70 (m, 3H), 1.60–1.54 (m, 2H), 1.48–1.42 (m, 2H), 1.23 (s, 24H), 0.86 (t, *J* = 6.8 Hz, 3H); ^13^C NMR (100 MHz, CDCl_3_) δ 160.0, 83.2, 56.6, 46.1, 33.9, 31.9, 29.8, 29.67 (3C), 29.64 (3C), 29.57, 29.3, 28.4, 28.3, 28.0, 22.7, 14.1; IR (CHCl_3_) ν_max_ 3255, 2919, 2852, 1723, 1469, 1248 (cm^−1^); HRMS (FAB) calcd. for C_20_H_38_NO_2_ ([M + H]^+^), 324.2903; found, 324.2903.

#### 3.1.13. (3a*S*,6*R*,6a*R*)-6-Hexadecylhexahydro-2*H*-cyclopenta[*d*]oxazol-2-one (**11c**)

A white solid (122 mg, 50%). m.p. 91.9–92.1 °C; ${\left[\text{α}\right]}_{\text{D}}^{20}$ +7.91 (c 0.46, CHCl_3_); ^1^H NMR (400 MHz, CDCl_3_) δ 5.51 (brs, 1H), 4.89 (t, *J* = 6.1 Hz, 1H), 4.23 (t, *J* = 6.5 Hz, 1H), 1.77–1.70 (m, 3H), 1.61–1.55 (m, 2H), 1.48–1.40 (m, 2H), 1.23 (s, 28H), 0.86 (t, *J* = 6.8 Hz, 3H); ^13^C NMR (100 MHz, CDCl_3_) δ 159.9, 83.2, 56.6, 46.1, 33.9, 31.9, 29.8, 29.68 (5C), 29.65 (3C), 29.58, 29.3, 28.4, 28.3, 28.0, 22.7, 14.1; IR (CHCl_3_) ν_max_ 3262, 2918, 2851, 1725, 1469, 1245 (cm^−1^); HRMS (FAB) calcd. for C_22_H_42_NO_2_ ([M + H]^+^), 352.3216; found, 352.3216.

#### 3.1.14. General Procedure for the Preparation of **4**

To a solution of **11** (0.071 mmol) in EtOH (1 mL) was added 1 M aq KOH (1 mL) at room temperature. The reaction mixture was stirred for 20 h at 85 °C. After the reaction mixture was cooled to room temperature, the solvent was removed under reduced pressure. The crude product was purified by column chromatography on silica gel (CH_2_Cl_2_/MeOH/NH_4_OH, 100:10:1) to give the desired product, **4**.

#### 3.1.15. (1*R*,2*S*,5*R*)-2-Amino-5-dodecylcyclopentan-1-ol (**4a**)

A white solid (17 mg, 88%). m.p. 93.2–96.2 °C; ${\left[\text{α}\right]}_{\text{D}}^{20}$ +3.96 (c 0.36, EtOH); ^1^H NMR (400 MHz, CDCl_3_) δ 3.68 (t, *J* = 3.5 Hz, 1H), 3.30 (td, *J* = 3.8 Hz, 8.4 Hz, 1H), 2.10 (brs, 2H), 1.91–1.86 (m, 1H), 1.74–1.66 (m, 2H), 1.56–1.34 (m, 1H), 1.23 (s, 23H), 0.86 (t, *J* = 6.8 Hz, 3H); ^13^C NMR (100 MHz, CDCl_3_) δ 74.4, 55.4, 44.2, 31.9, 30.9, 29.95, 29.93, 29.68 (3C), 29.64, 29.3, 28.5, 28.0, 22.7, 14.1; IR (CHCl_3_) ν_max_ 3054, 2922, 2852, 2727, 1469, 992 (cm^−1^); HRMS (FAB) calcd. for C_17_H_36_NO ([M + H]^+^), 270.2797; found, 270.2794.

#### 3.1.16. (1*R*,2*S*,5*R*)-2-Amino-5-tetradecylcyclopentan-1-ol (**4b**)

A white solid (19 mg, 90%). m.p. 100.1–101.0 °C; ${\left[\text{α}\right]}_{\text{D}}^{20}$ +3.29 (c 0.25, EtOH); ^1^H NMR (400 MHz, CDCl_3_) δ 3.70 (t, *J* = 3.4 Hz, 1H), 3.30 (td, *J* = 3.9 Hz, 8.5 Hz, 1H), 2.44 (brs, 3H), 1.91–1.85 (m, 1H), 1.75–1.65 (m, 2H), 1.54–1.34 (m, 1H), 1.23 (s, 27H), 0.86 (t, *J* = 6.8 Hz, 3H); ^13^C NMR (100 MHz, CDCl_3_) δ 74.4, 55.4, 44.1, 31.9, 30.8, 30.0, 29.9, 29.69 (5C), 29.65 (2C), 29.3, 28.5, 28.0, 22.7, 14.1; IR (CHCl_3_) ν_max_ 3049, 2921, 2852, 2729, 1469, 994 (cm^−1^); HRMS (FAB) calcd. for C_19_H_40_NO ([M + H]^+^), 298.3110; found, 298.3117.

#### 3.1.17. (1*R*,2*S*,5*R*)-2-Amino-5-hexadecylcyclopentan-1-ol (**4c**)

A white solid (21 mg, 91%). m.p. 102.1–103.0 °C; ${\left[\text{α}\right]}_{\text{D}}^{20}$ +1.89 (c 0.51, CHCl_3_); ^1^H NMR (400 MHz, CDCl_3_) δ 3.70 (t, *J* = 3.5 Hz, 1H), 3.30 (td, *J* = 3.8 Hz, 8.2 Hz, 1H), 2.41 (brs, 3H), 1.89–1.85 (m, 1H), 1.75–1.65 (m, 2H), 1.54–1.34 (m, 1H), 1.23 (s, 31H), 0.86 (t, *J* = 6.8 Hz, 3H); ^13^C NMR (100 MHz, CDCl_3_) δ 74.5, 55.5, 44.2, 31.9, 30.9, 30.0, 29.9, 29.69 (7C), 29.65 (2C), 29.4, 28.5, 28.0, 22.7, 14.1; IR (CHCl_3_) ν_max_ 3057, 2921, 2852, 2728, 1468, 995 (cm^−1^); HRMS (FAB) calcd. for C_21_H_44_NO ([M + H]^+^), 326.3423; found, 326.3433.

### 3.2. Biological Evaluation

#### 3.2.1. Sulforhodamine B (SRB) Assay

Cells (5 × 10^4^ cells/mL) were treated with various concentrations of test compounds in 96-well culture plates for 72 h. After incubation, cells were fixed with 10% trichloroacetic acid (TCA), dried and stained with 0.4% SRB in 1% acetic acid. The unbound dye was washed out, and the stained cells were dried and resuspended in 10 mM Tris (pH 10.0). The absorbance at 515 nm was measured, and cell proliferation was determined as follows: cell proliferation (%) = (average absorbance_compound_ − average absorbance_day zero_)/(average absorbance_control_ − average absorbance_day zero_) × 100. IC_50_ values were calculated by nonlinear regression analysis using TableCurve 2D v5.01 (Systat Software Inc., Richmond, CA, USA).

#### 3.2.2. Sphingosine Kinase Inhibition Assay

In 384-well polystyrene plates, Sphk1 and Sphk2 (BPS Bioscience, San Diego, CA, USA) were incubated in kinase buffer (5 mM MOPS, pH 7.2, 2.5 mM β-glycerol-phosphate, 5 mM MgCl_2_, 1 mM EGTA, 0.4 mM EDTA, 0.5 mM DTT) containing 1 µM sphingosine, ATP (50 µM for SphK1; 150 µM for SphK2) and test compounds with a final concentration of 1% DMSO for 1 h at room temperature. The amount of ATP transferred was measured with the ADP-Glo™ kinase assay kit (Catalog #V9101, Promega, Madison, WI, USA) according to the manufacturer’s instructions. IC_50_ values were calculated using GraphPad Prism 5 software (GraphPad Software Inc., La Jolla, CA, USA).

### 3.3. Molecular Modeling

To understand the binding mode of the pachastrissamine and its analogue, we performed a flexible docking study using the Schrödinger Glide program with standard precision settings (Schrödinger, LLC, New York, NY, USA, http://www.schrodinger.com). The X-ray crystal structure of the human SphK1 (PDB Code 3VZB) was obtained from the Protein Data Bank (PDB, [36]). The pachastrissamine and **4b** were minimized using a Merck Molecular Force Field (MMFF) with a dielectric constant of 80.0 using the MacroModel program suite. The initial structure of pachastrissamine and **4b** were built based on the sphingosine structure co-crystallized with SphK1. The best docking result was visualized using Discovery Studio 4.1 (Accelrys Software, Inc., San Diego, CA, USA). The hydrogen bonding interactions were displayed as green dashed lines between the ligand and the SphK1.

## 4. Conclusions

We have reported the synthesis and biological evaluation of the carbocyclic analogues of pachastrissamine with varying chain lengths. The designed carbocyclic analogues were efficiently synthesized in good overall yield by the tandem ene/yne-ene metathesis as a key step. Among them, the analogue **4b** exhibits comparable cytotoxic activity and more potent inhibitory activity against sphingosine kinases, compared to the parent natural product. In our docking model, **4b** showed an additional interaction caused by the adjacent hydrophobic amino acid residue, which could explain its increased inhibitory activity. These results imply that bioisosteric replacement of the ethereal oxygen in the cyclic core to the methylene carbon is possible. Our findings are valuable in the development of sphingosine kinase inhibitors, which may lead to the development of promising new anti-cancer therapeutic agents. Further studies toward the biochemical and pharmacological properties of the carbocyclic analogue, based on these findings, are worthy of pursuing in the future.

## Supplementary Files

Supplementary File 1
