# Supplementary material for: Synthesis and Biological Evaluation of Carbocyclic Analogues of Pachastrissamine
Source: Mar Drugs. 2015 Feb 3;13(2):824–37. doi: 10.3390/md13020824 (PMC4344604; doi:10.3390/md13020824)
Supplement: Supplementary File 1 [file marinedrugs-13-00824-s001.pdf]

Supplementary Information

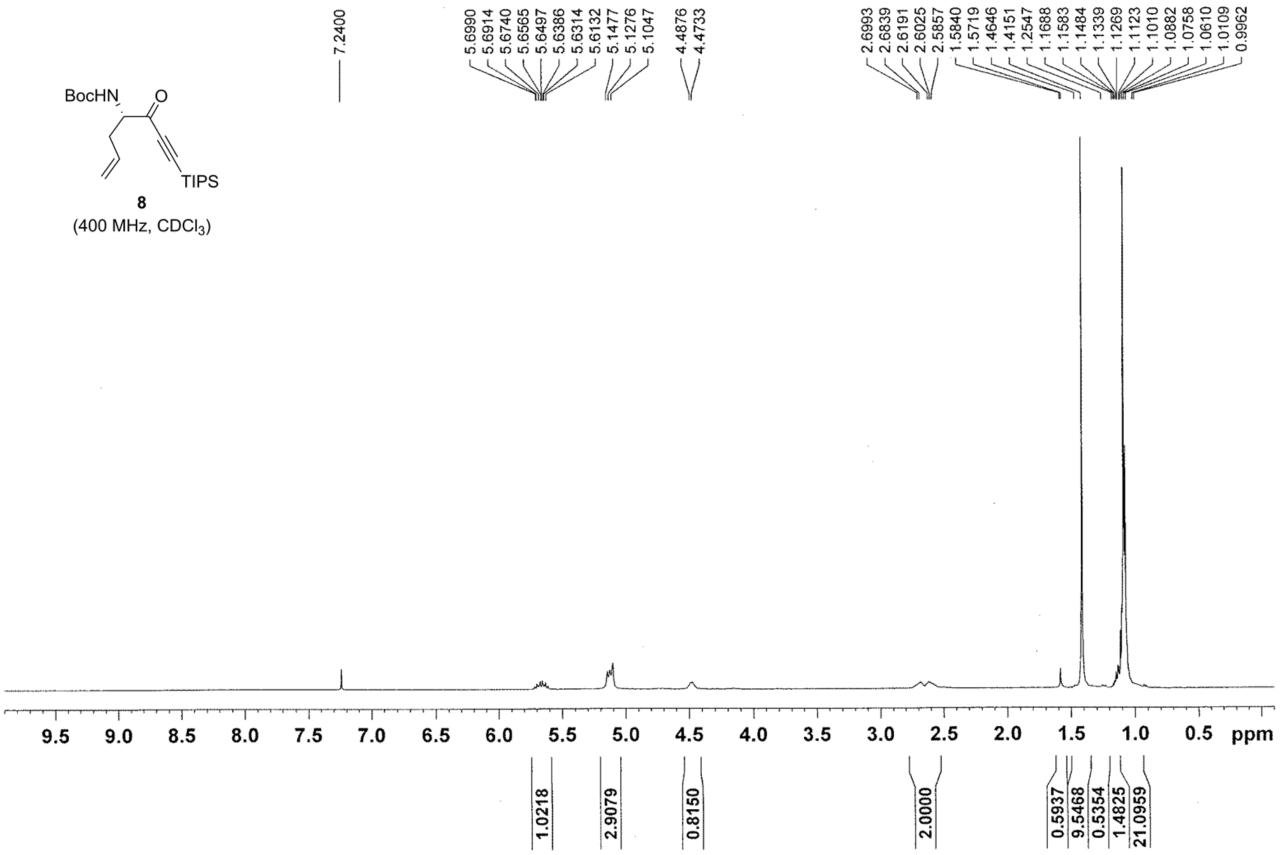

Figure S1. ^{1}H NMR spectrum of **8**.

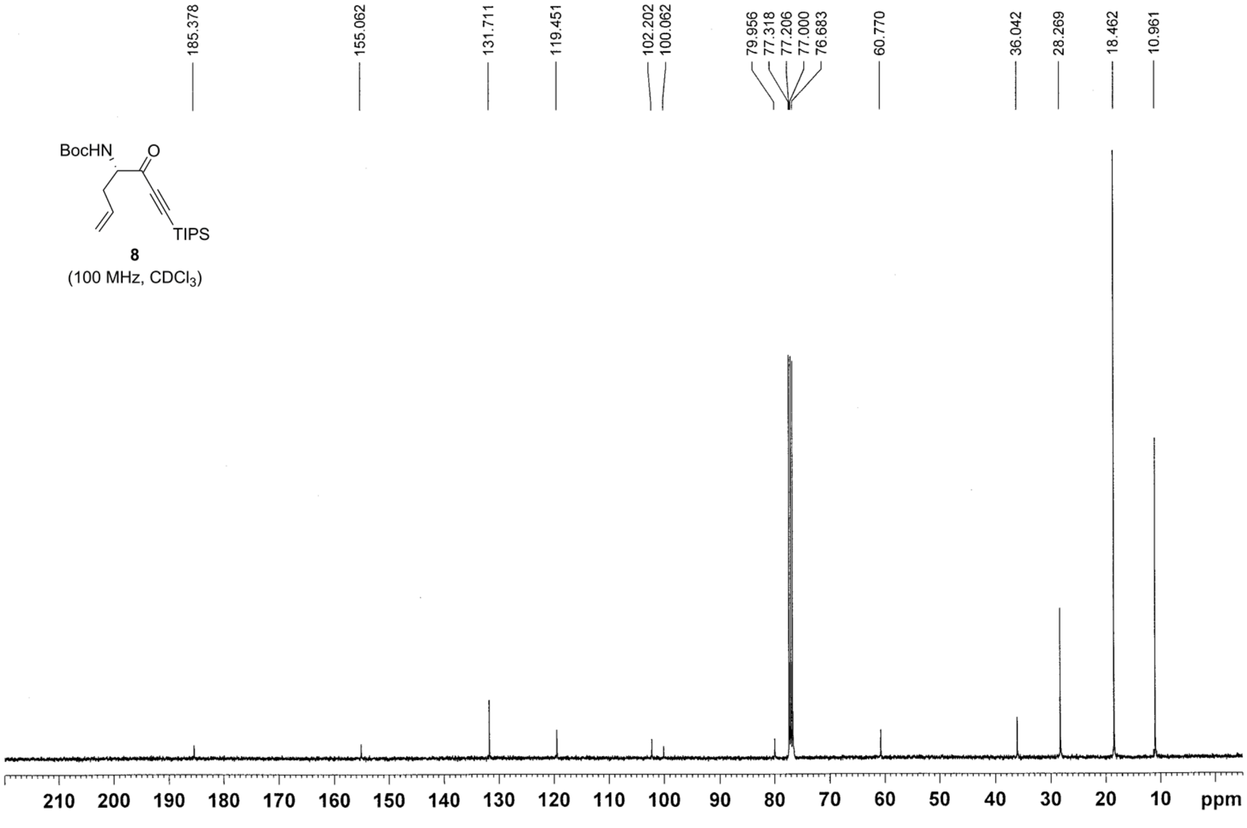

Figure S2. ^{13}C NMR spectrum of **8**.

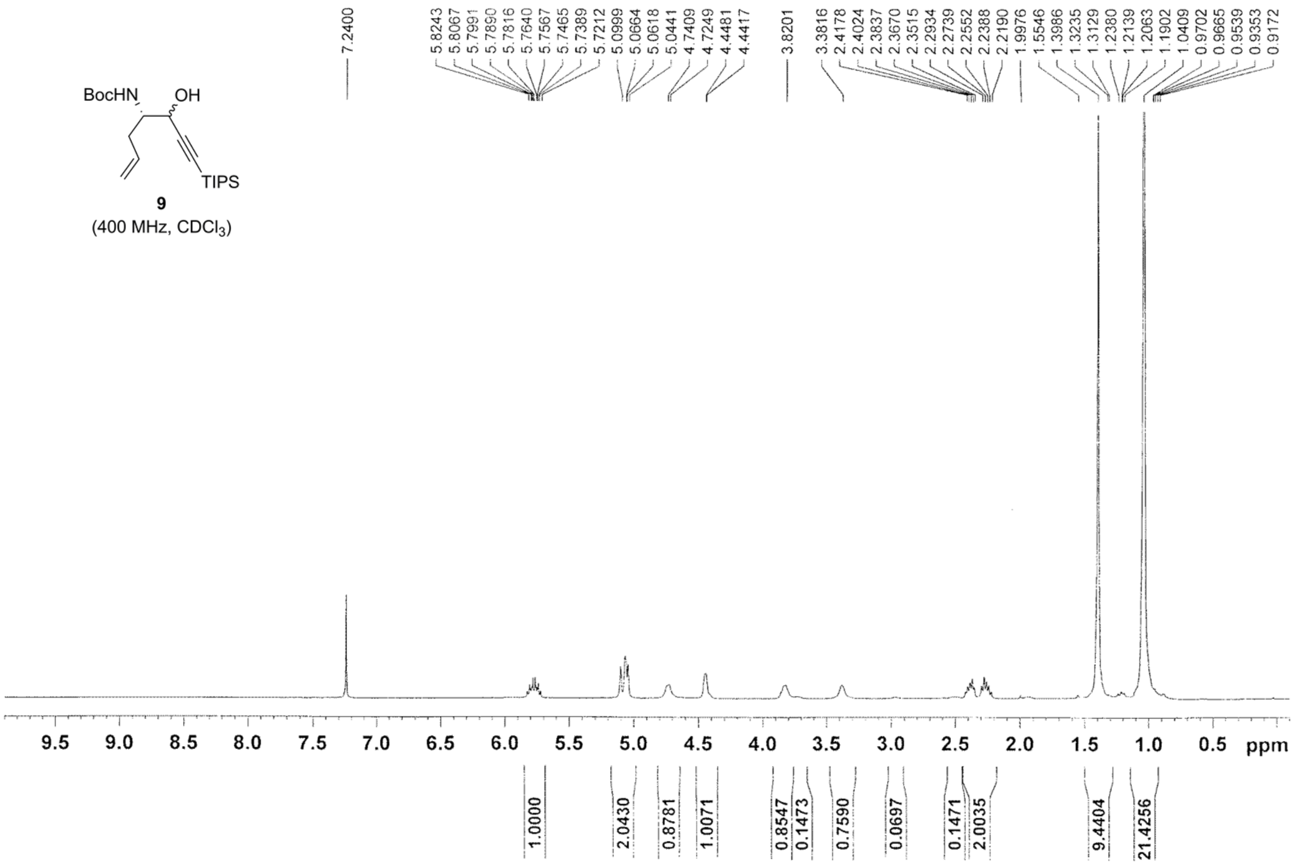

Figure S3. ^{1}H NMR spectrum of **9**.

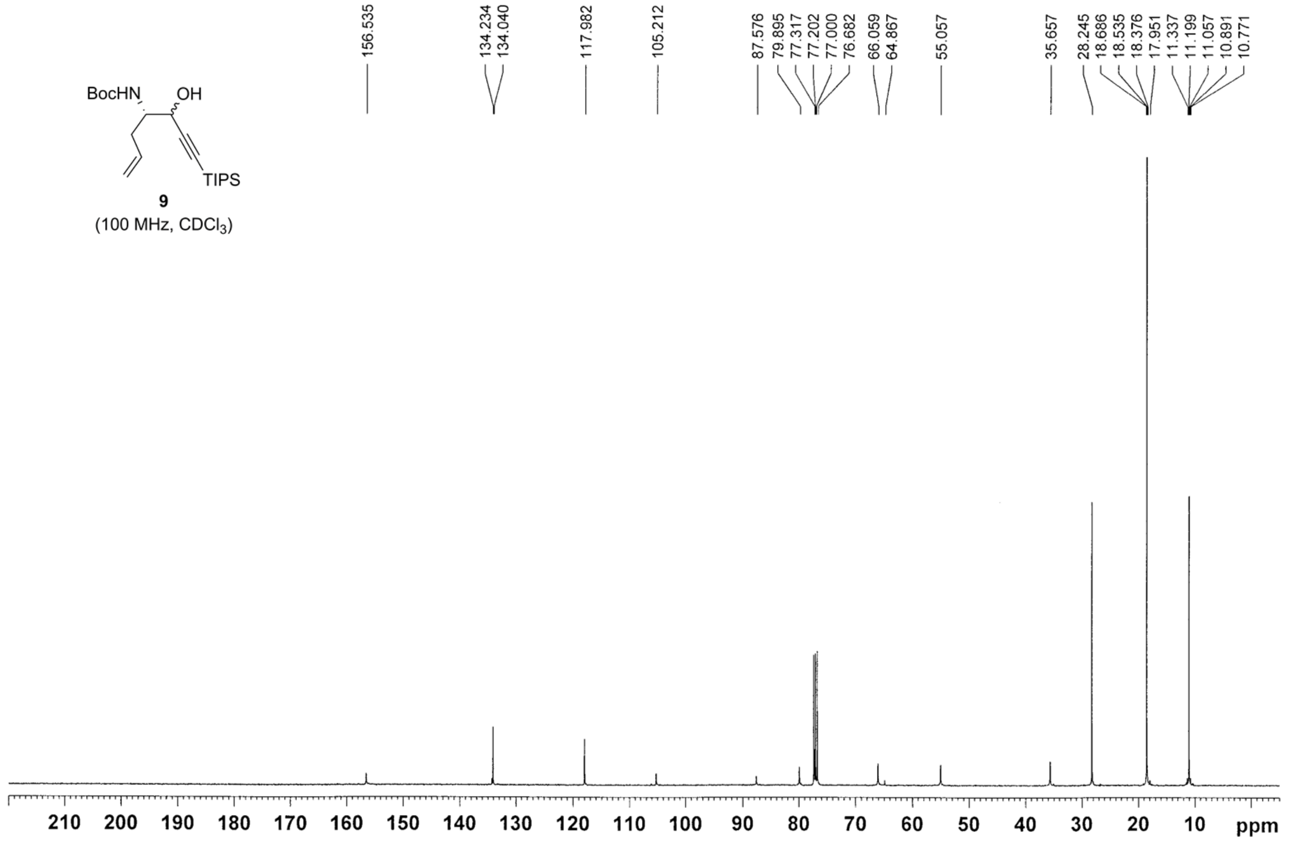

Figure S4. ^{13}C NMR spectrum of **9**.

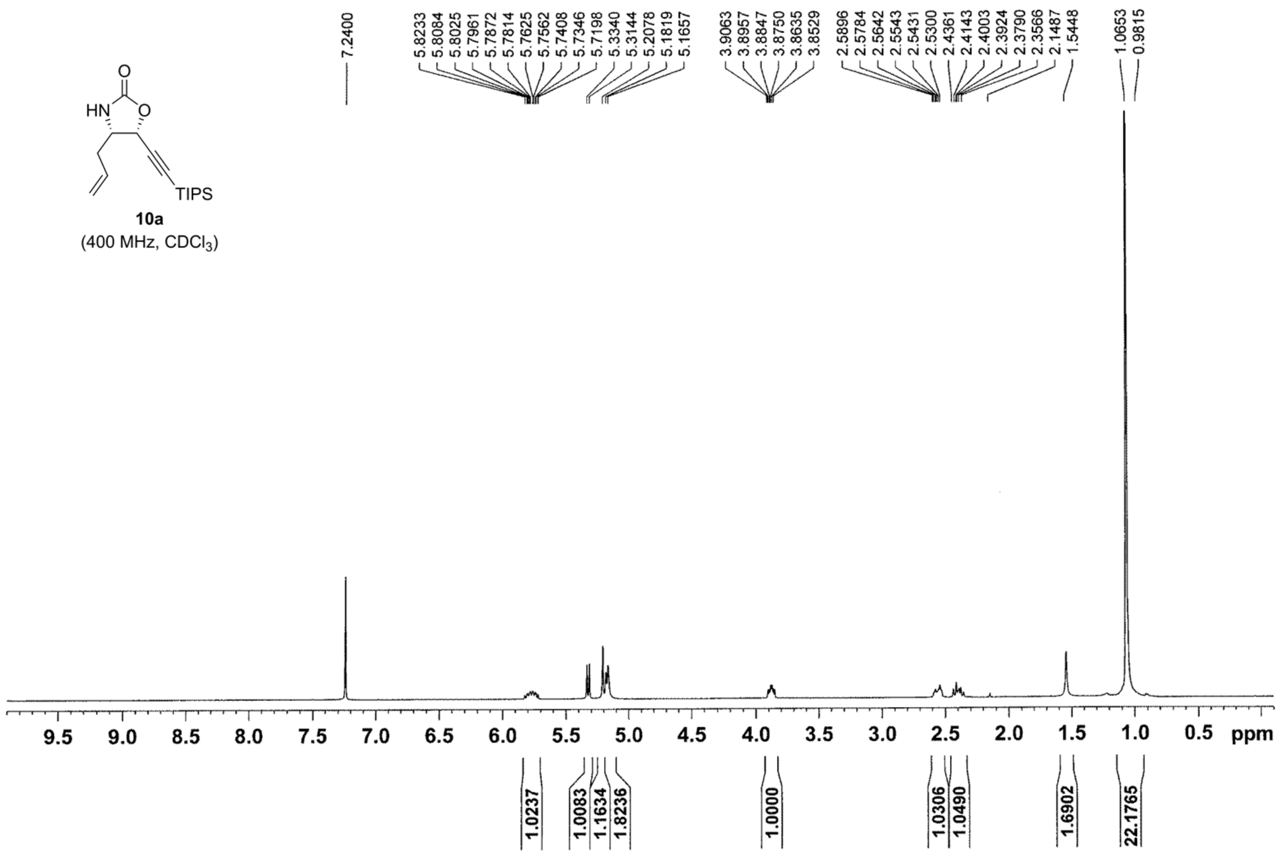

Figure S5. ^{1}H NMR spectrum of **10a**.

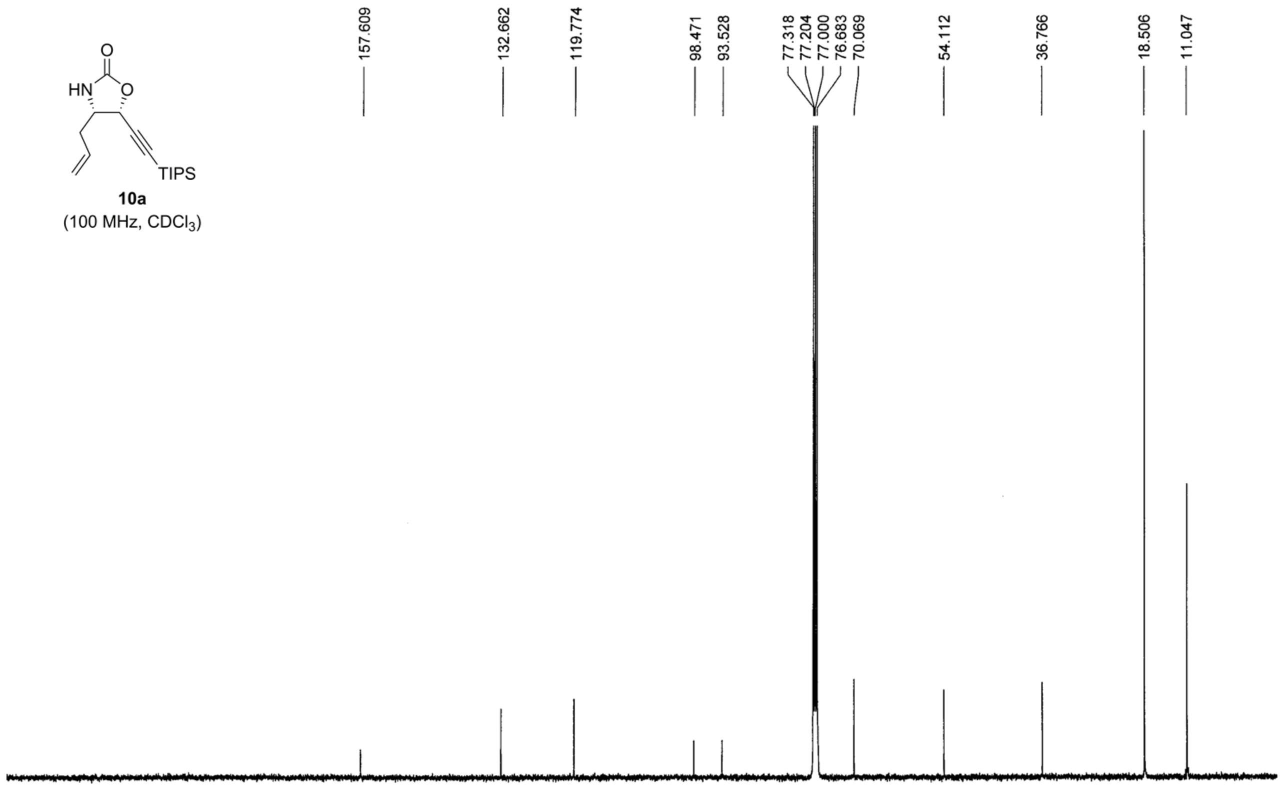

Figure S6. ^{13}C NMR spectrum of **10a**.

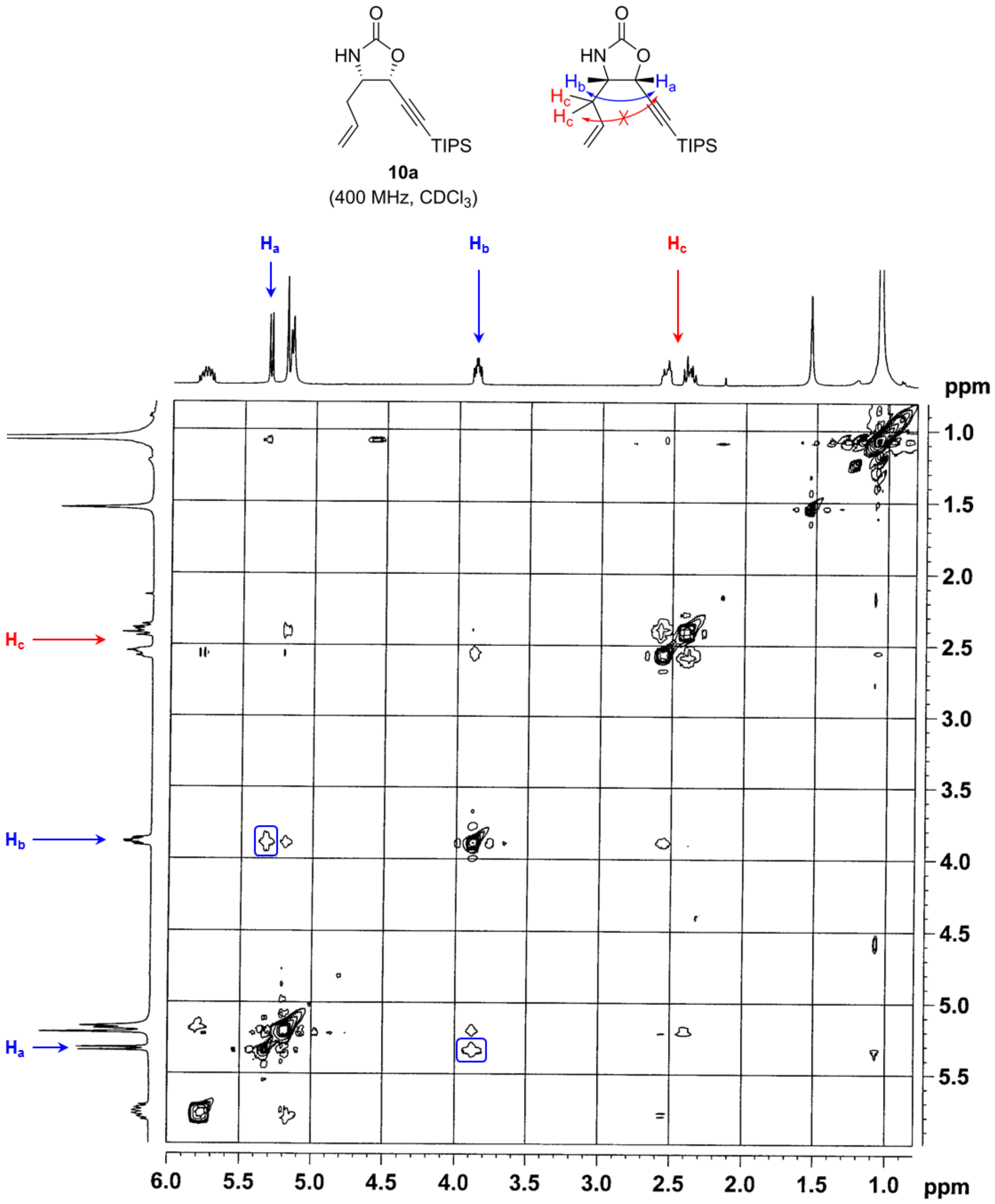

Figure S7. 2D NOESY spectrum of **10a**.

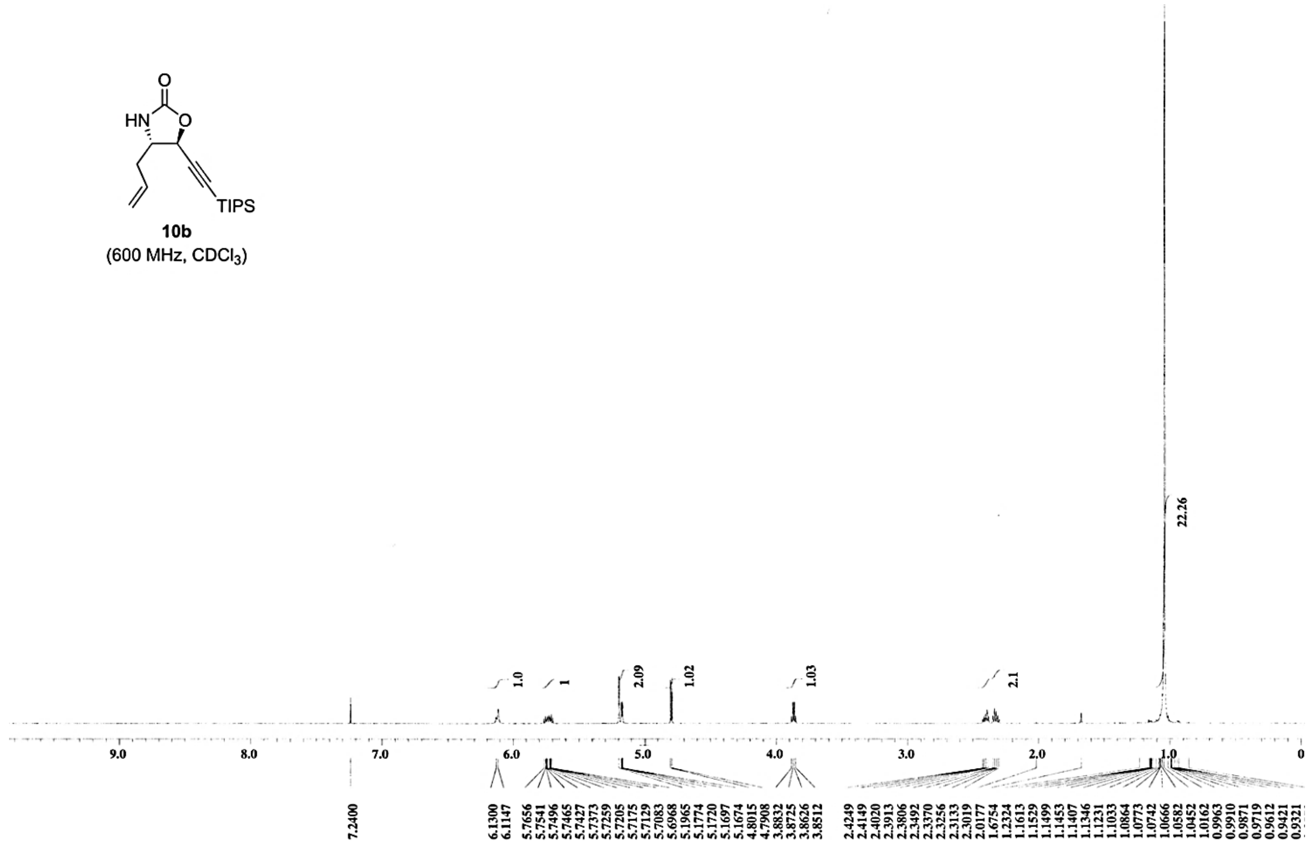

Figure S8. ^{1}H NMR spectrum of **10b**.

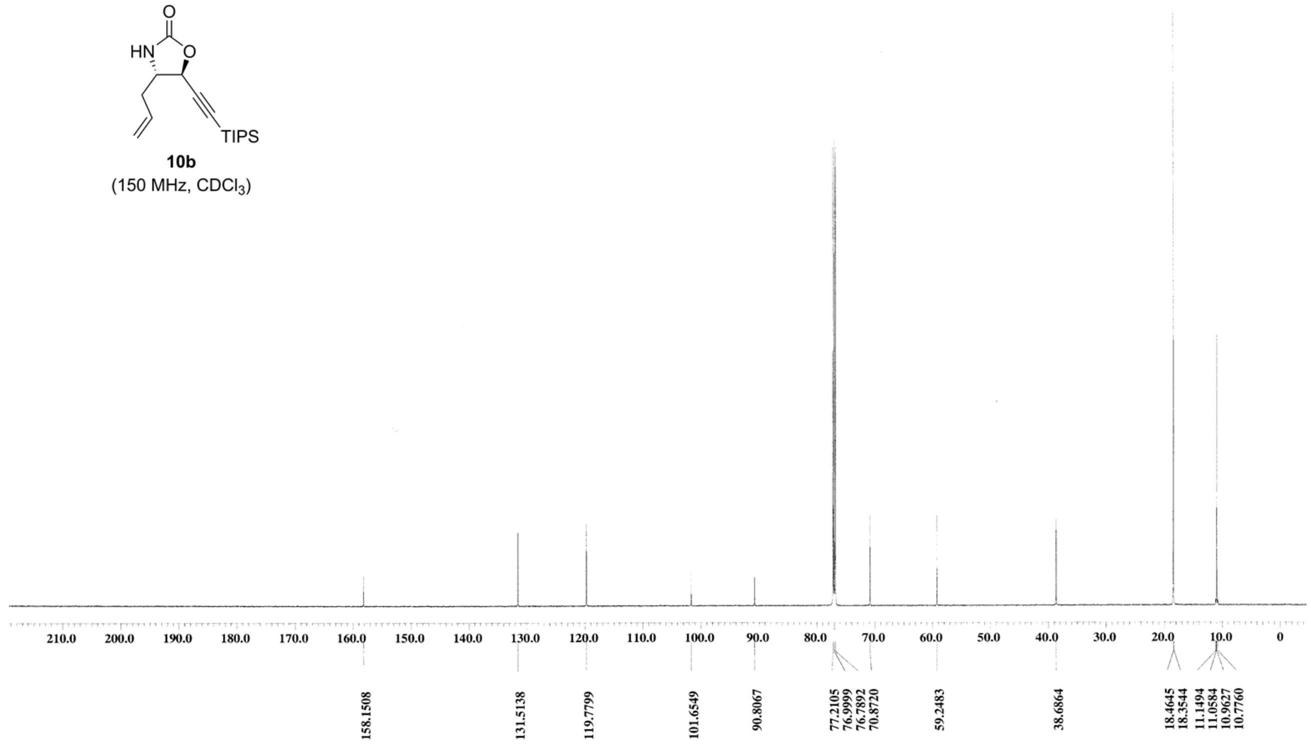

Figure S9. ^{13}C NMR spectrum of **10b**.

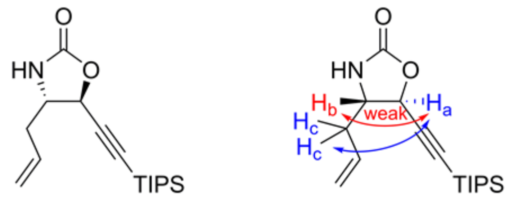

10b
(600 MHz, $CDCl_3$)

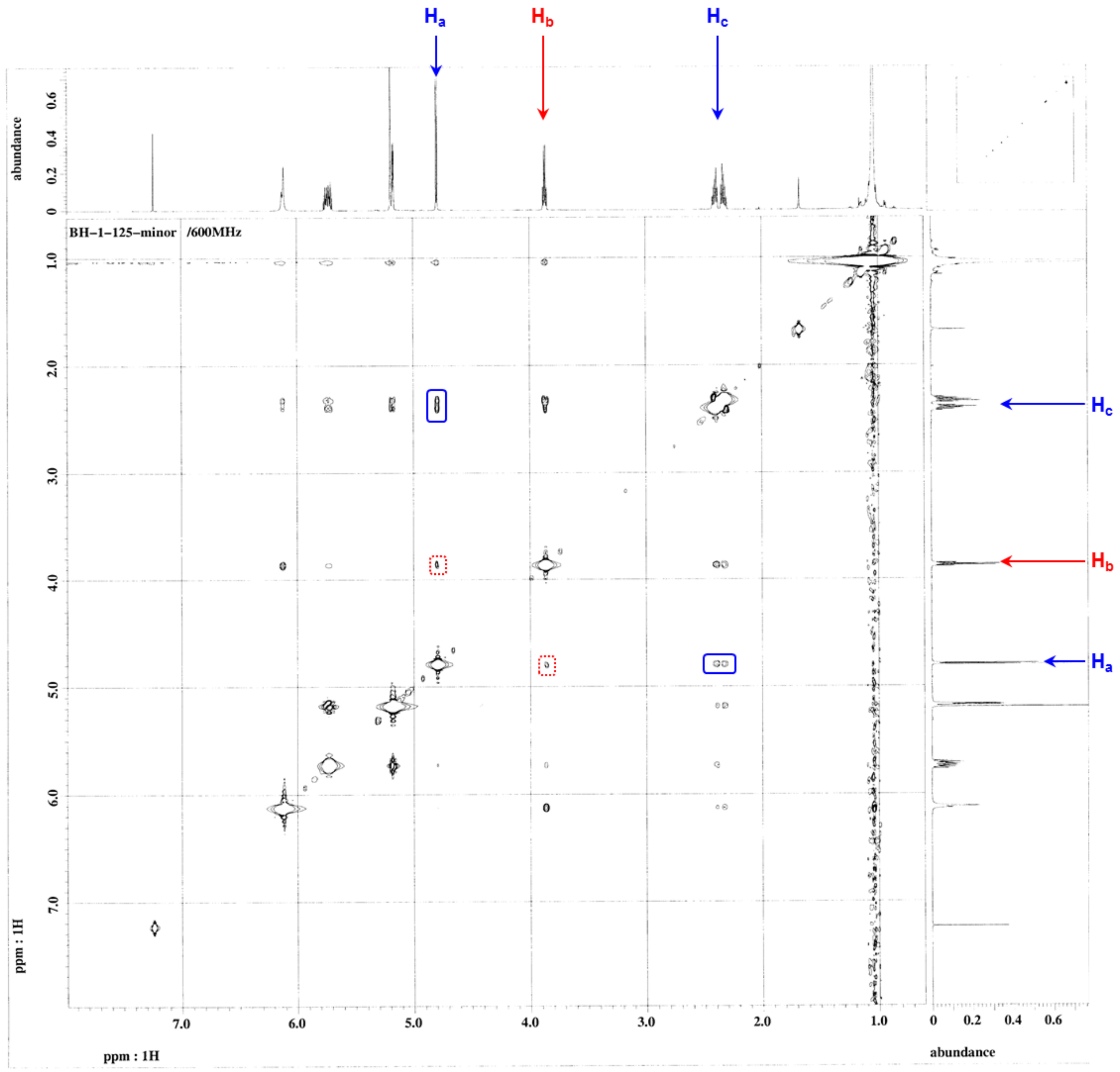

Figure S10. 2D NOESY spectrum of **10b**.

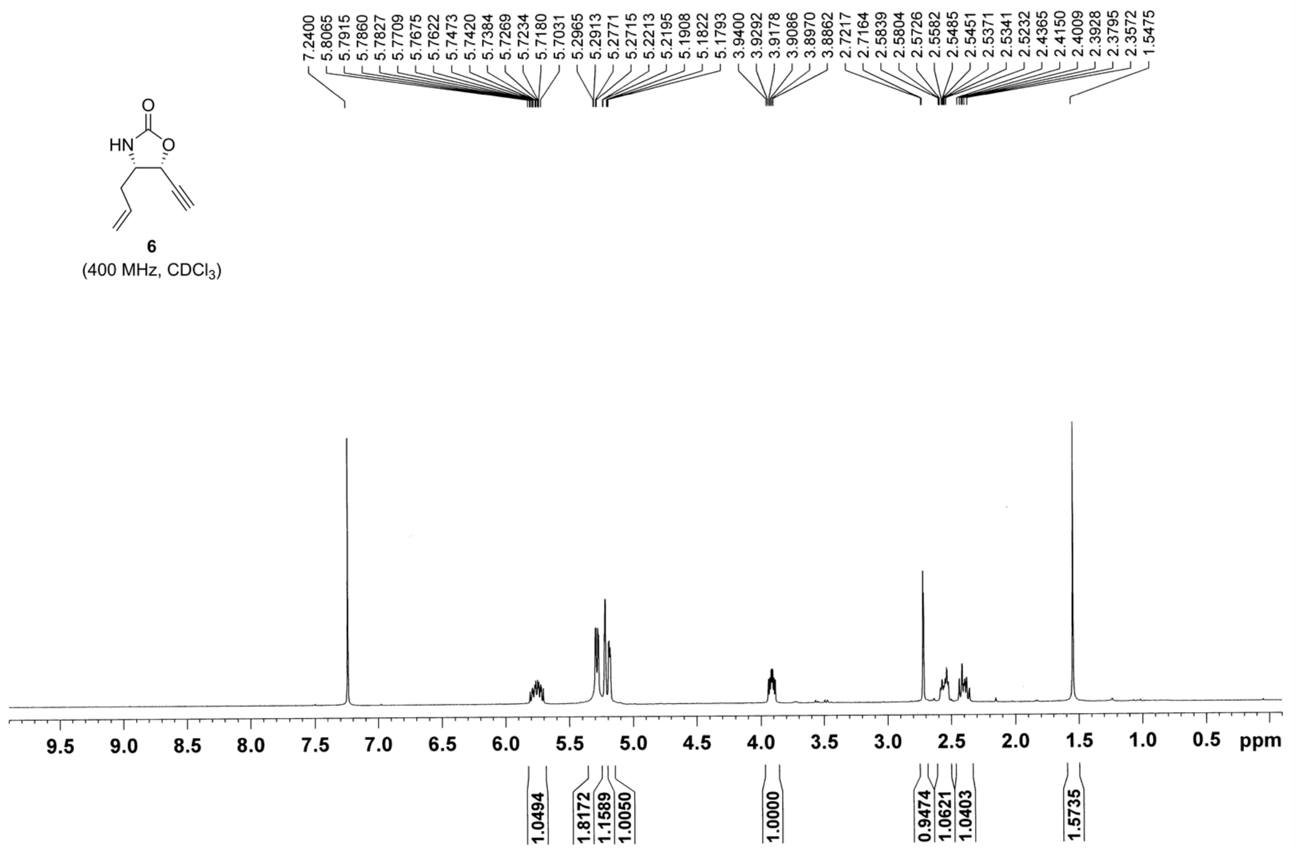

Figure S11. ^{1}H NMR spectrum of **6**.

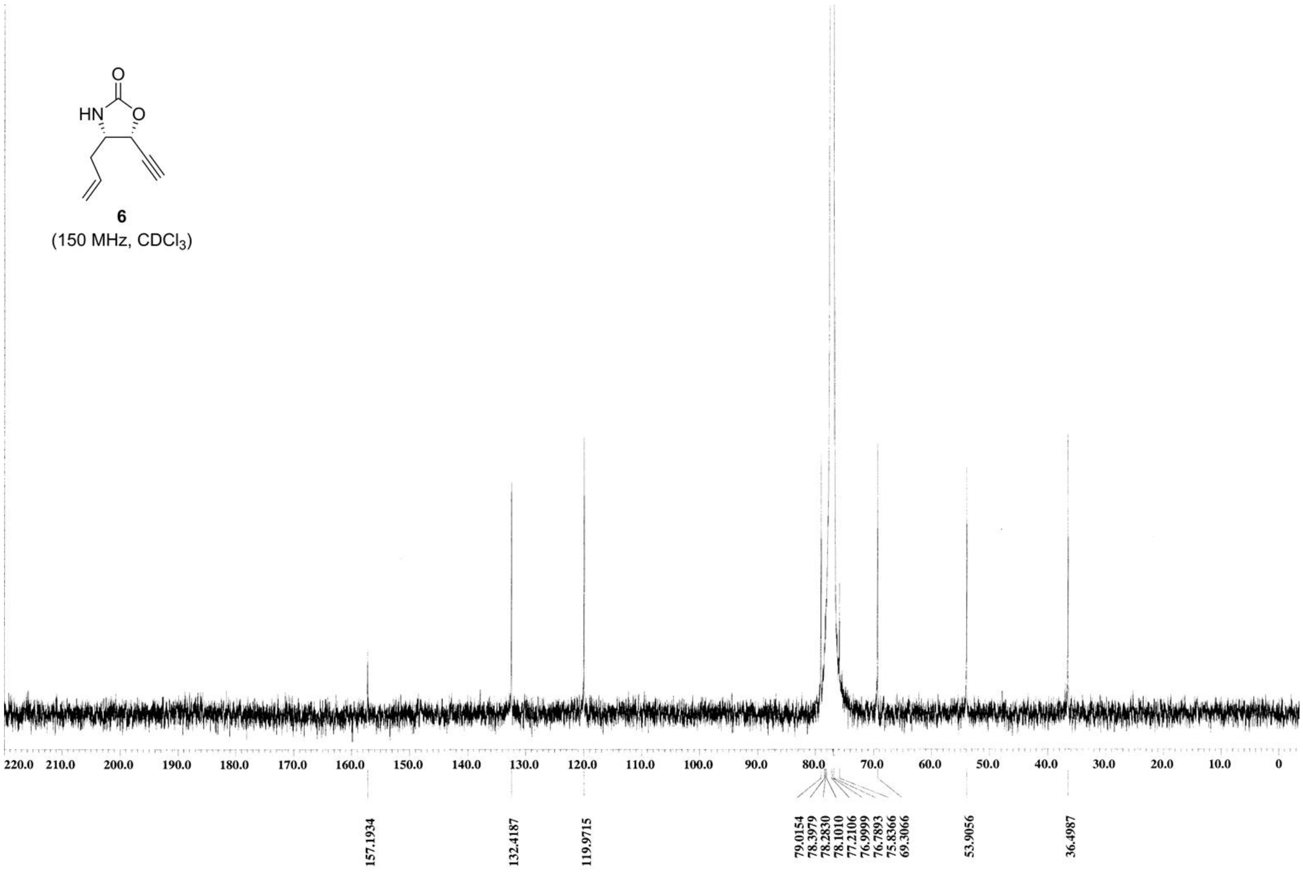

Figure S12. ^{13}C NMR spectrum of **6**.

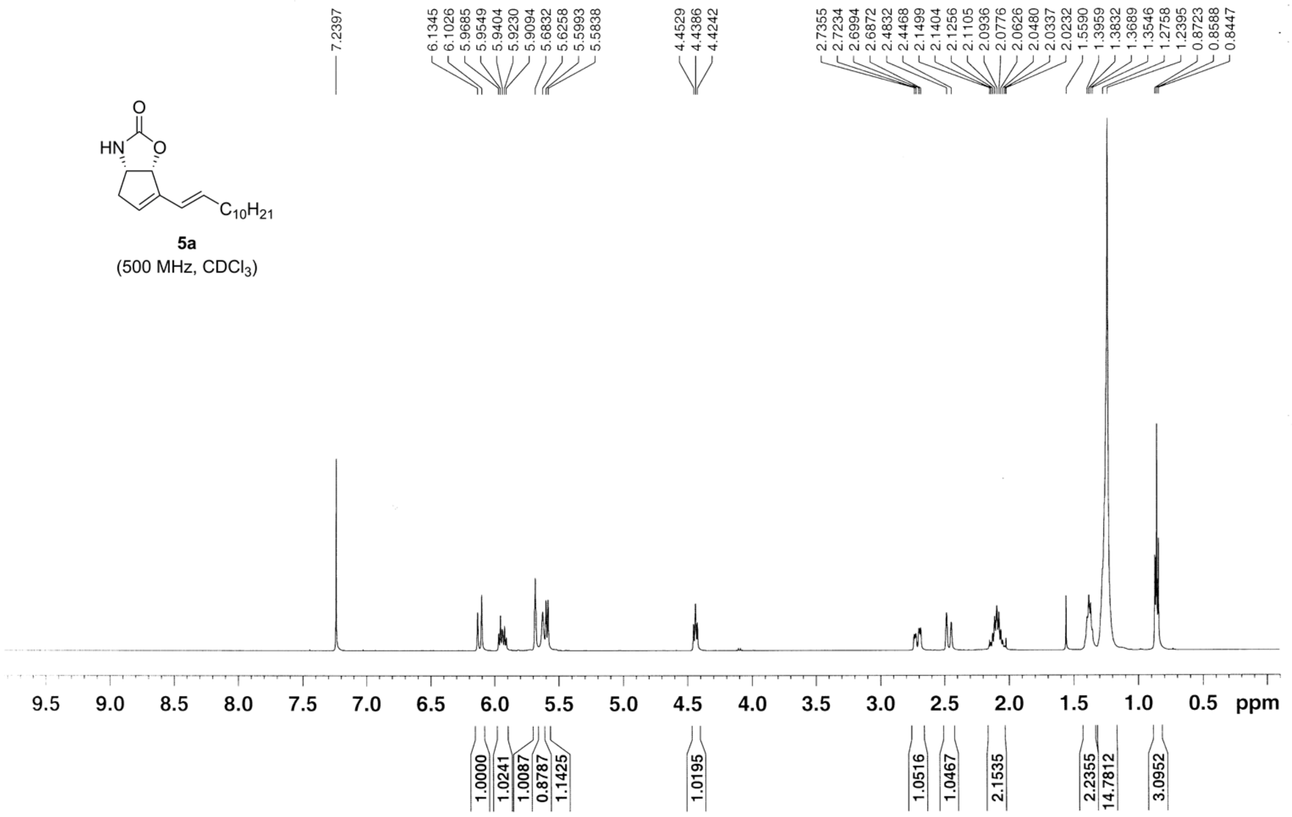

Figure S13. ^{1}H NMR spectrum of **5a**.

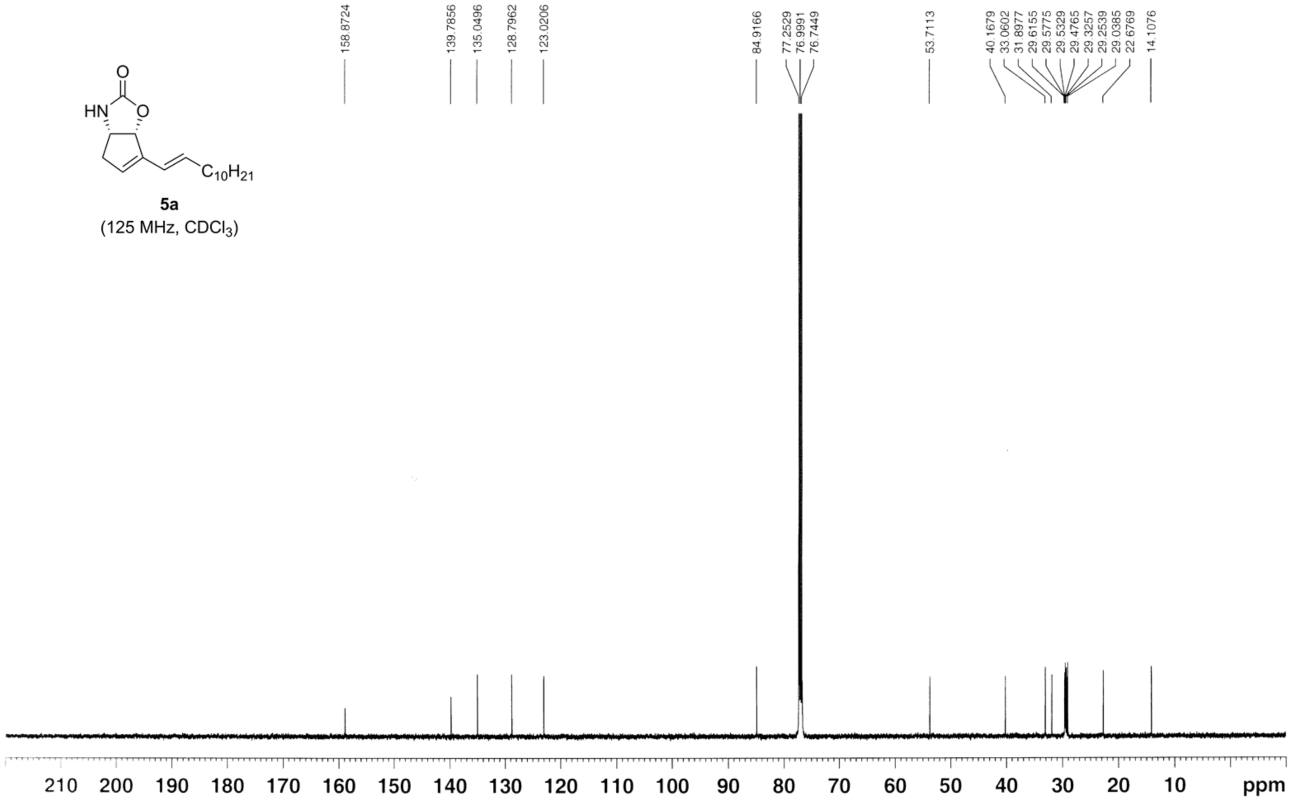

Figure S14. ^{13}C NMR spectrum of **5a**.

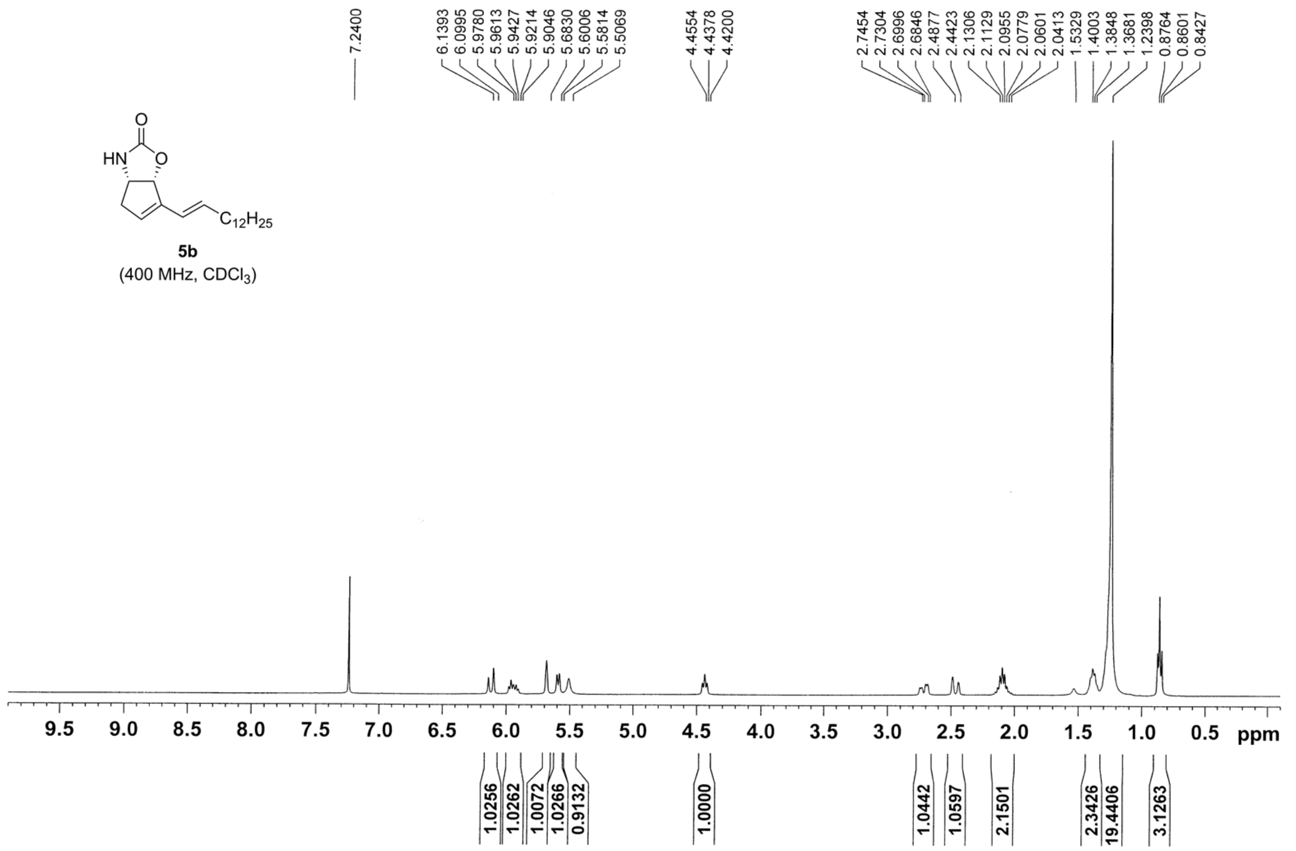

Figure S15. ^{1}H NMR spectrum of **5b**.

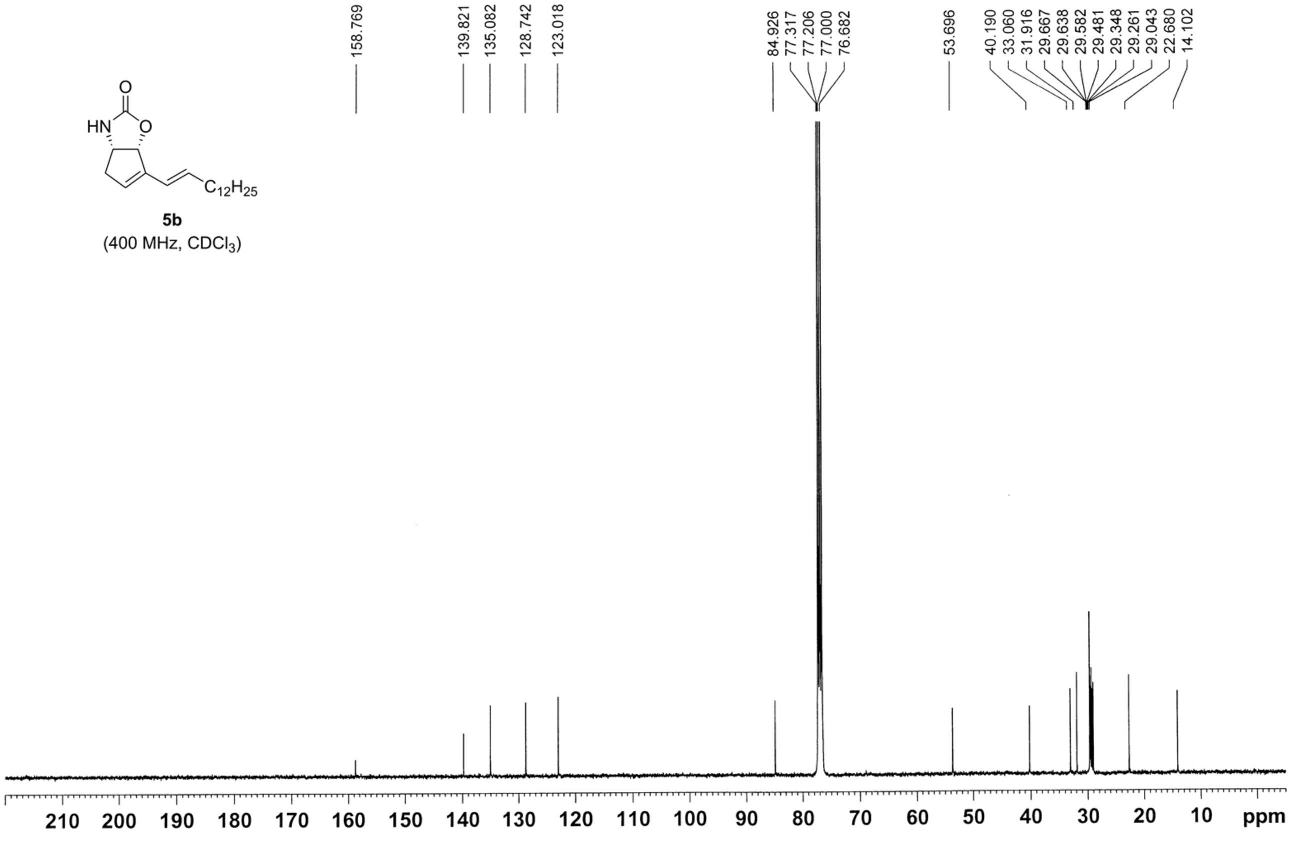

Figure S16. ^{13}C NMR spectrum of **5b**.

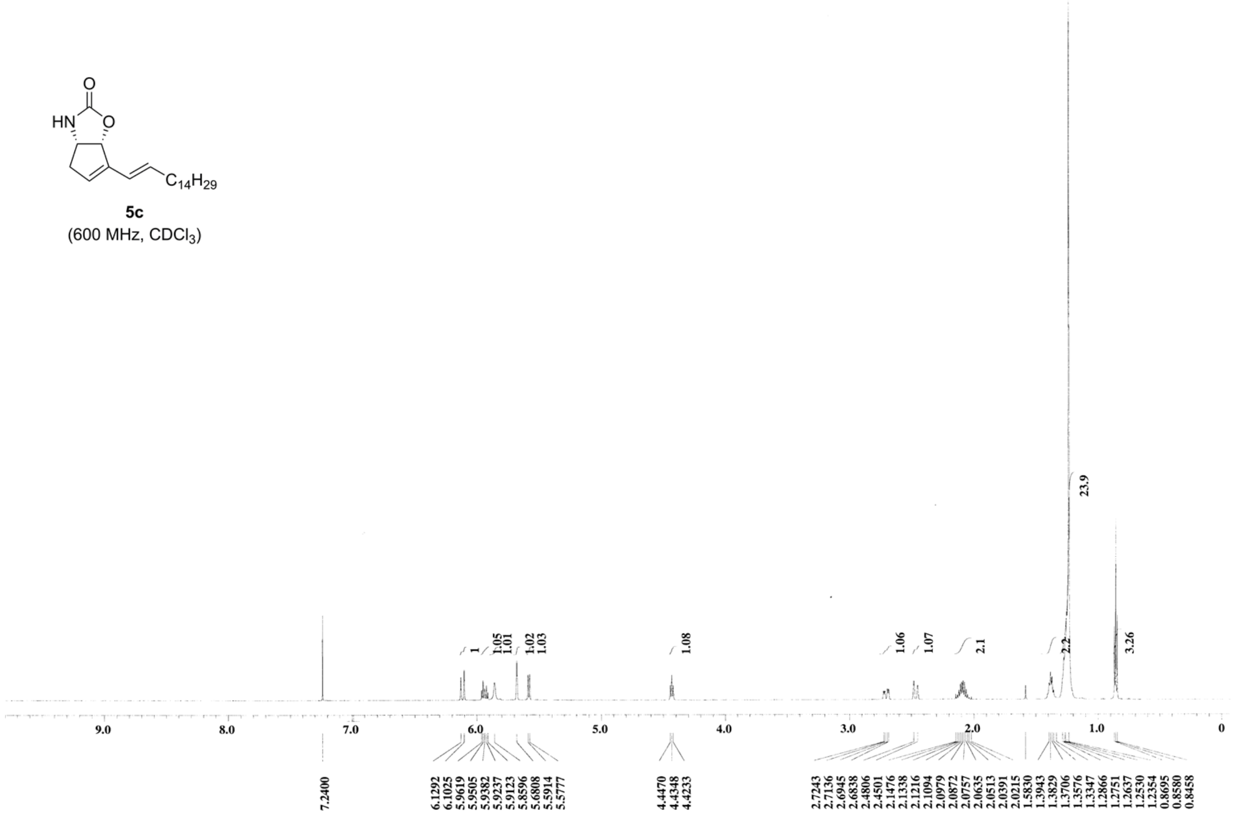

Figure S17. ^{1}H NMR spectrum of **5c**.

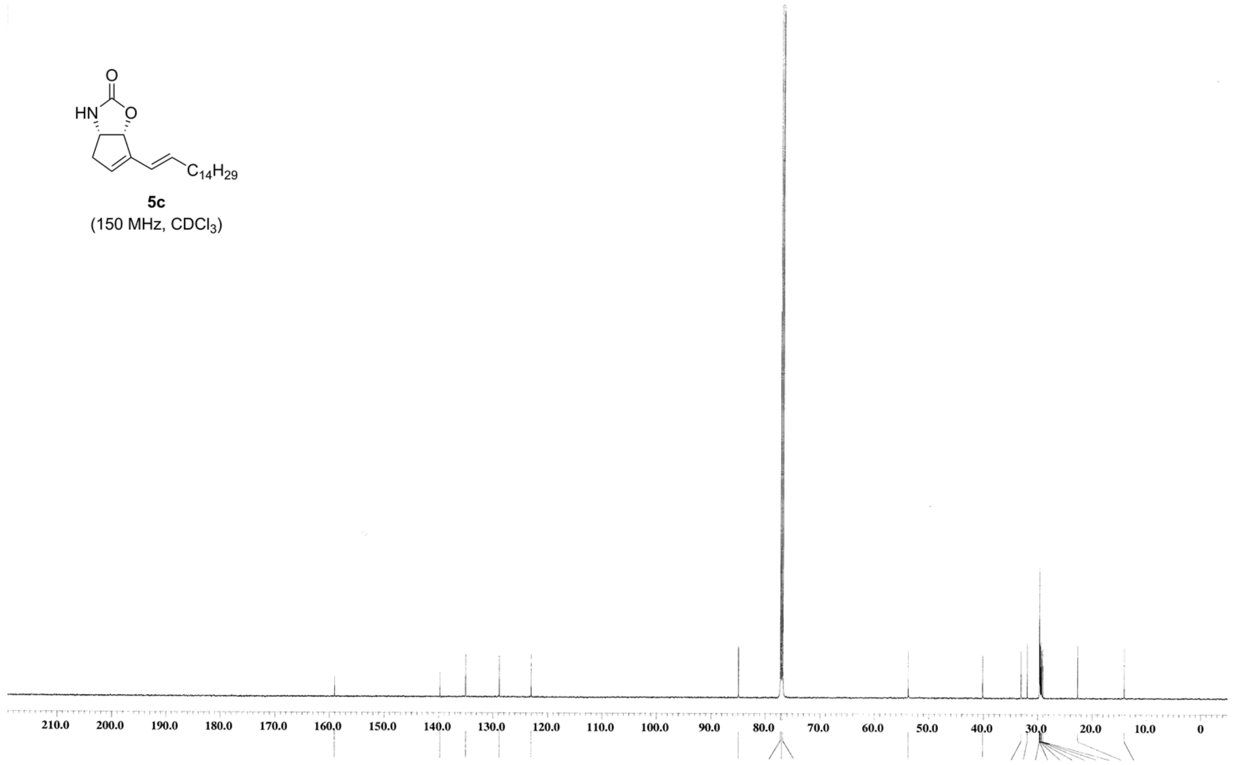

Figure S18. ^{13}C NMR spectrum of **5c**.

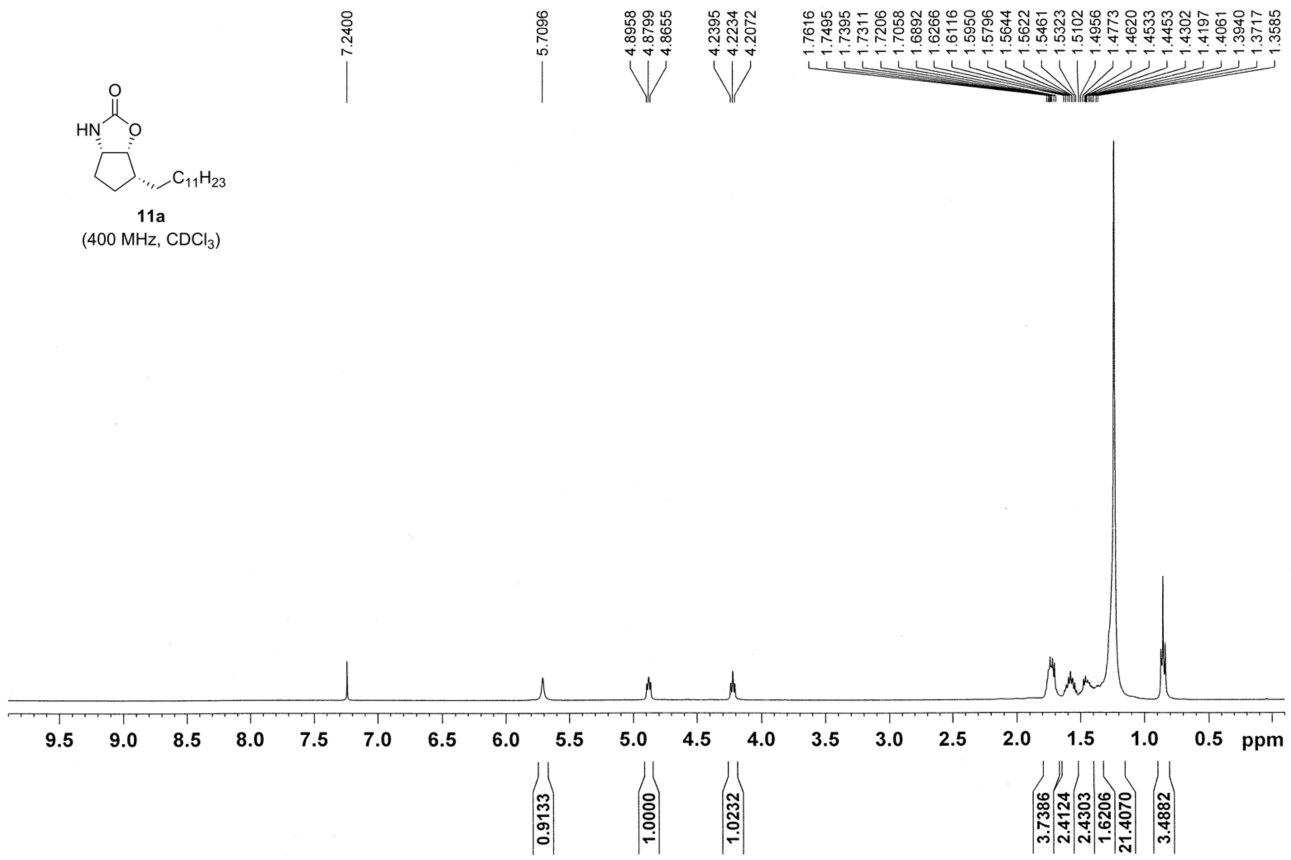

Figure S19. ^{1}H NMR spectrum of **11a**.

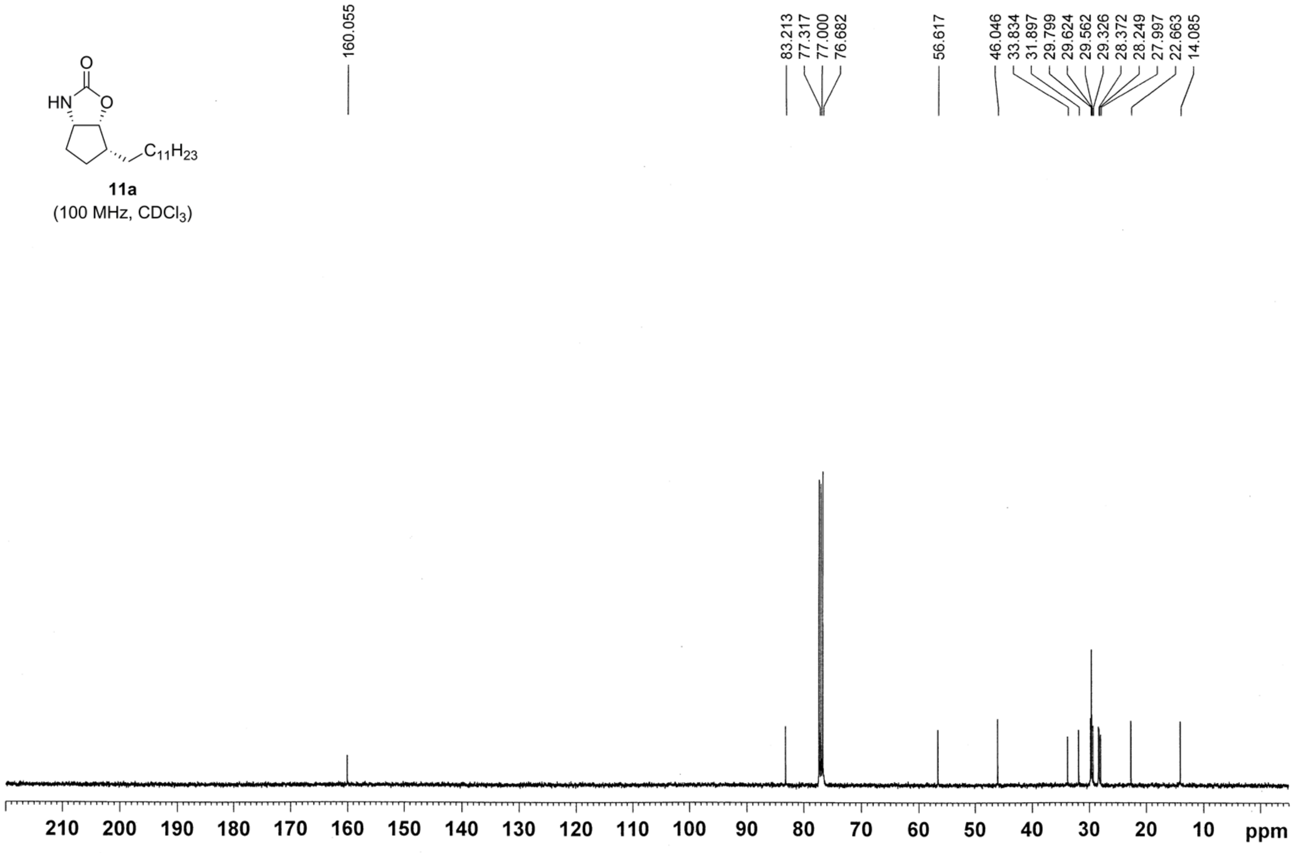

Figure S20. ^{13}C NMR spectrum of **11a**.

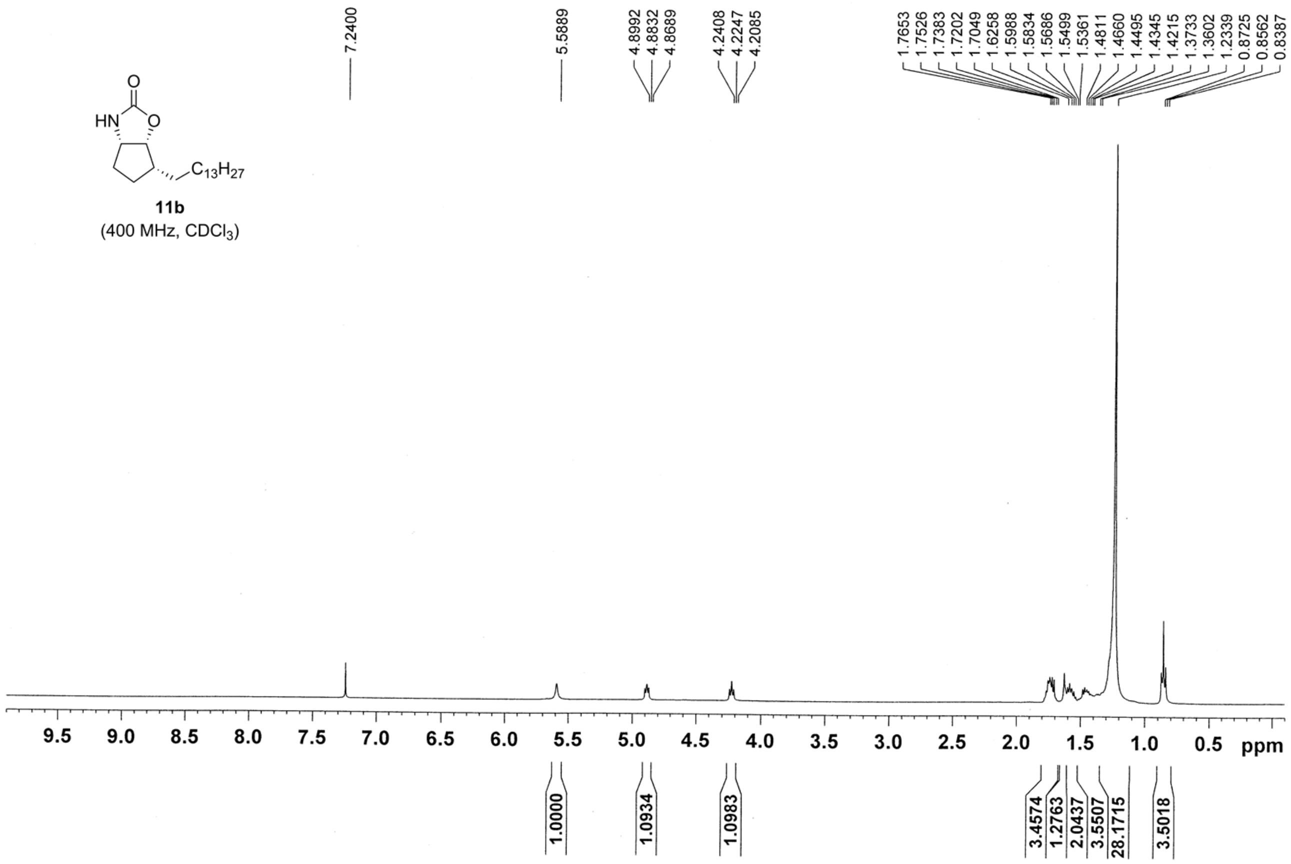

Figure S21. ^{1}H NMR spectrum of **11b**.

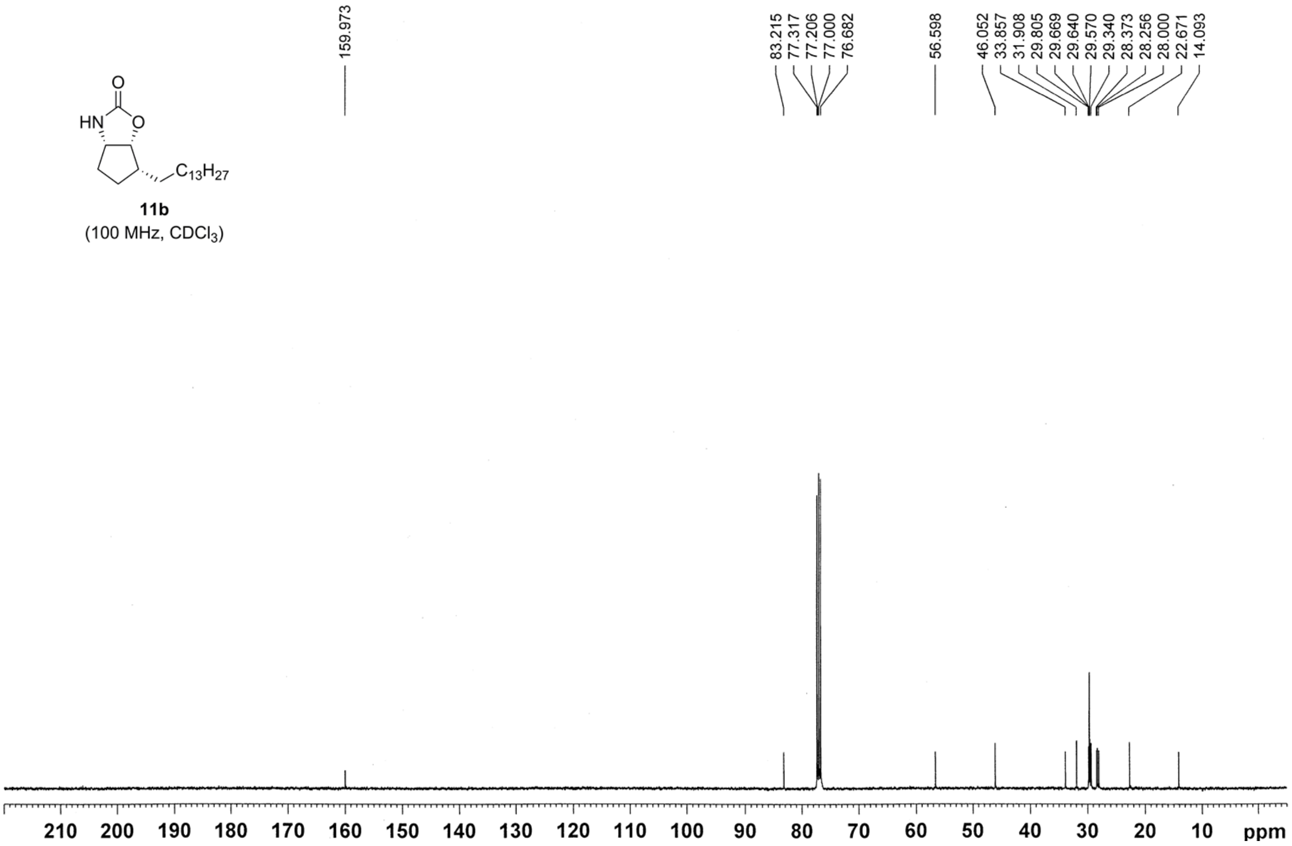

Figure S22. ^{13}C NMR spectrum of **11b**.

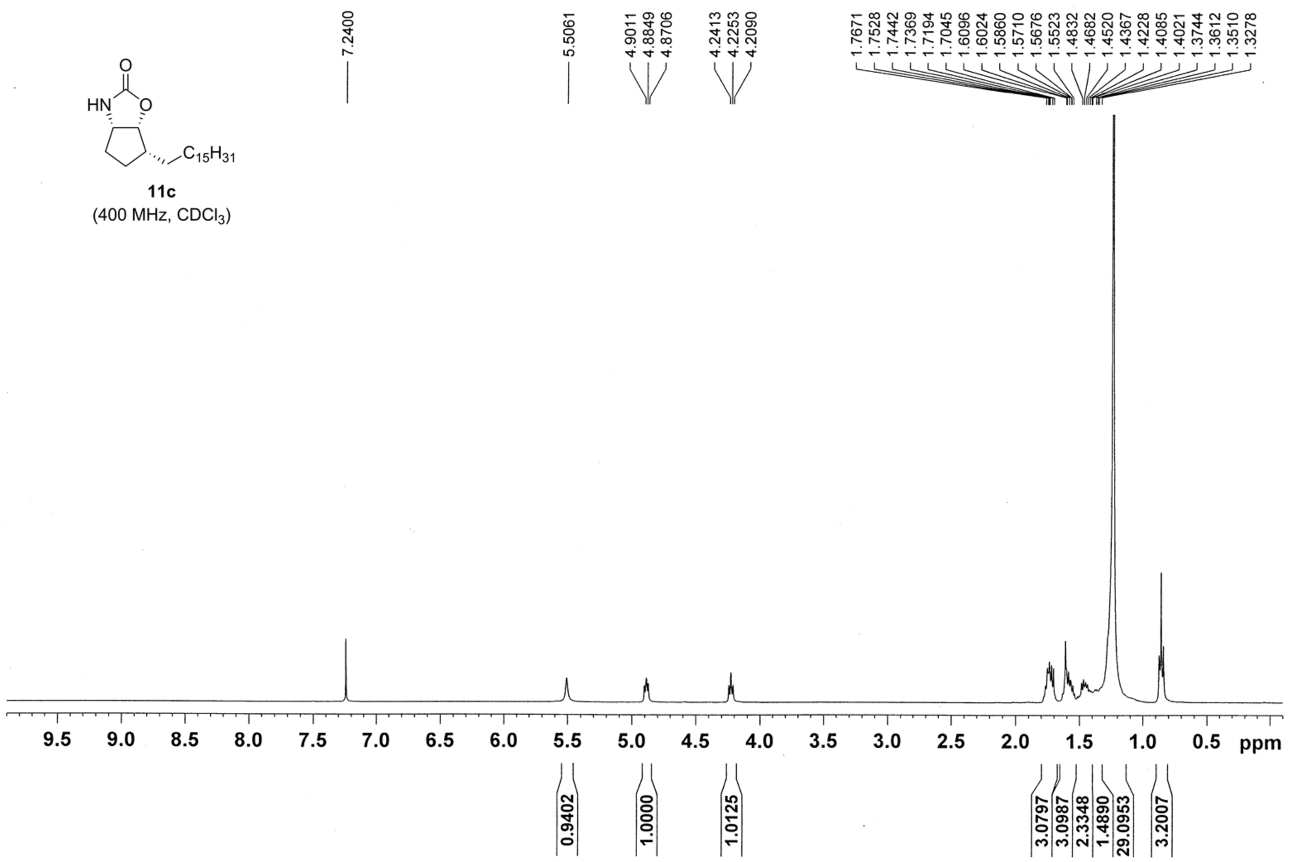

Figure S23. ^{1}H NMR spectrum of **11c**.

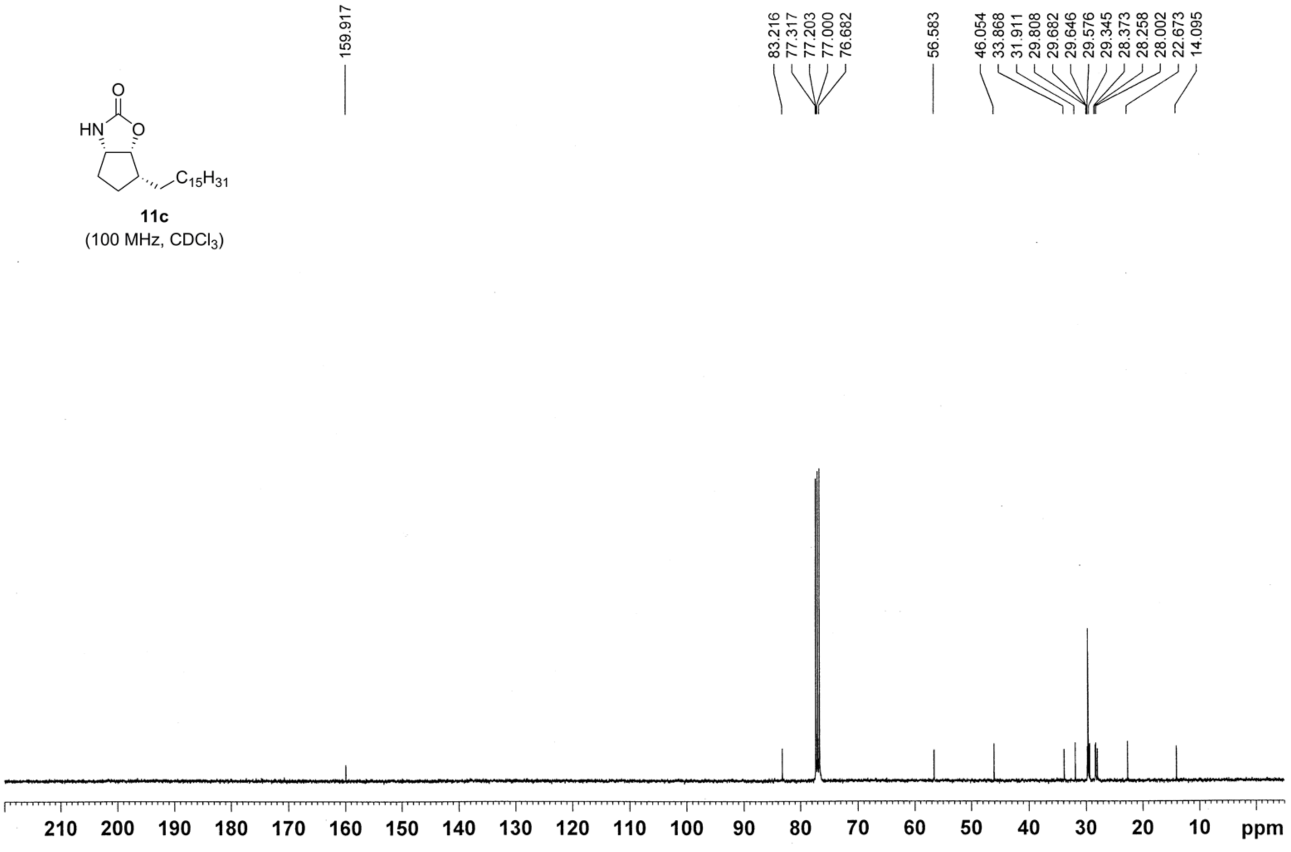

Figure S24. ^{13}C NMR spectrum of **11c**.

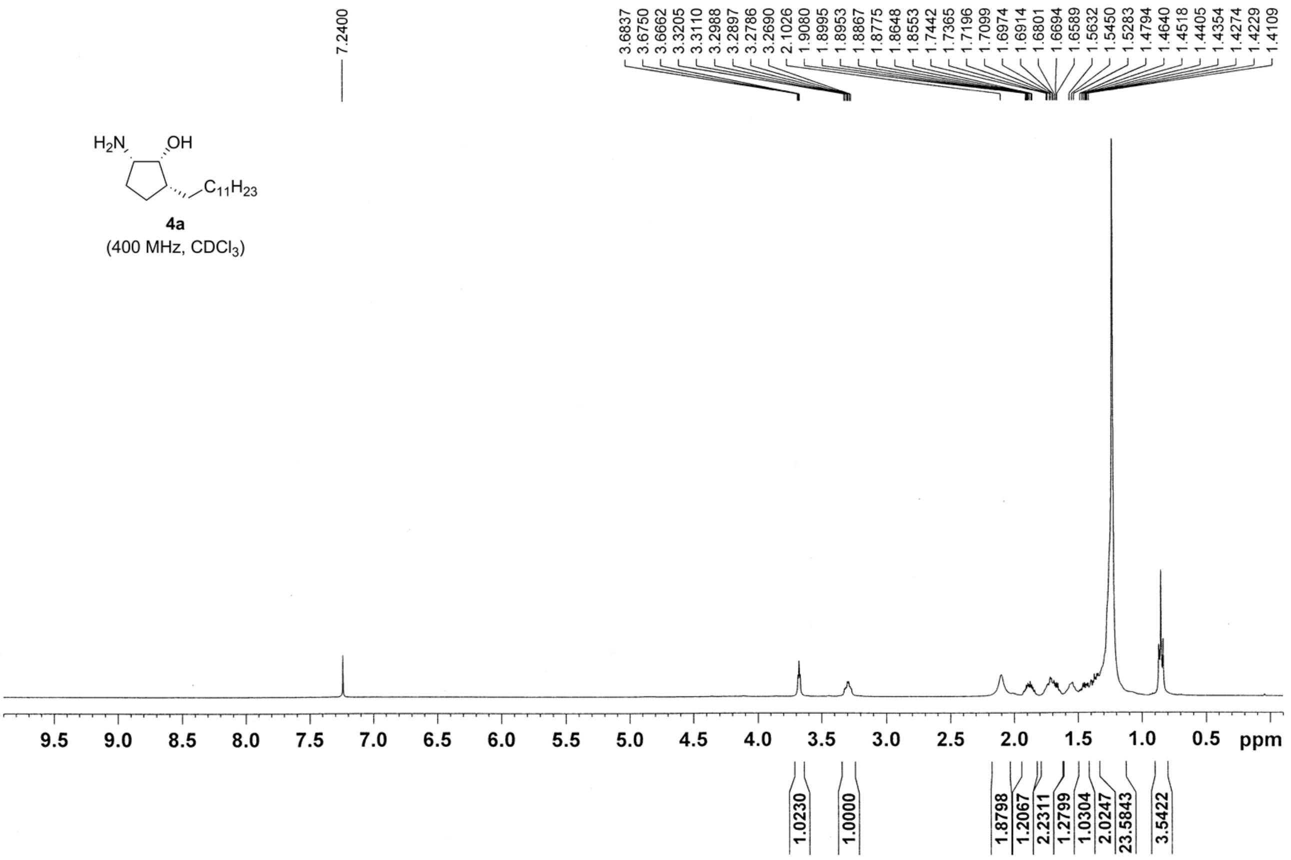

Figure S25. ^{1}H NMR spectrum of **4a**.

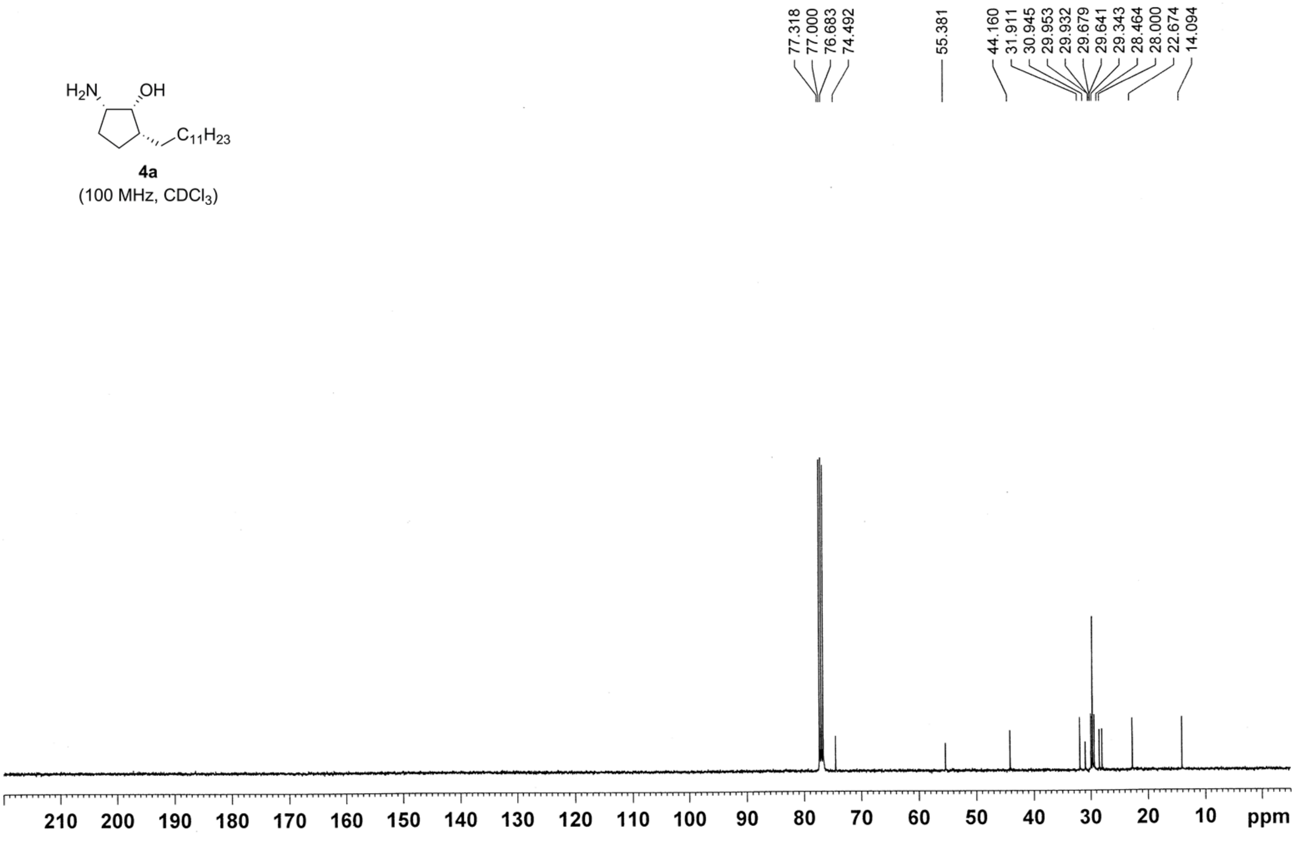

Figure S26. ^{13}C NMR spectrum of **4a**.

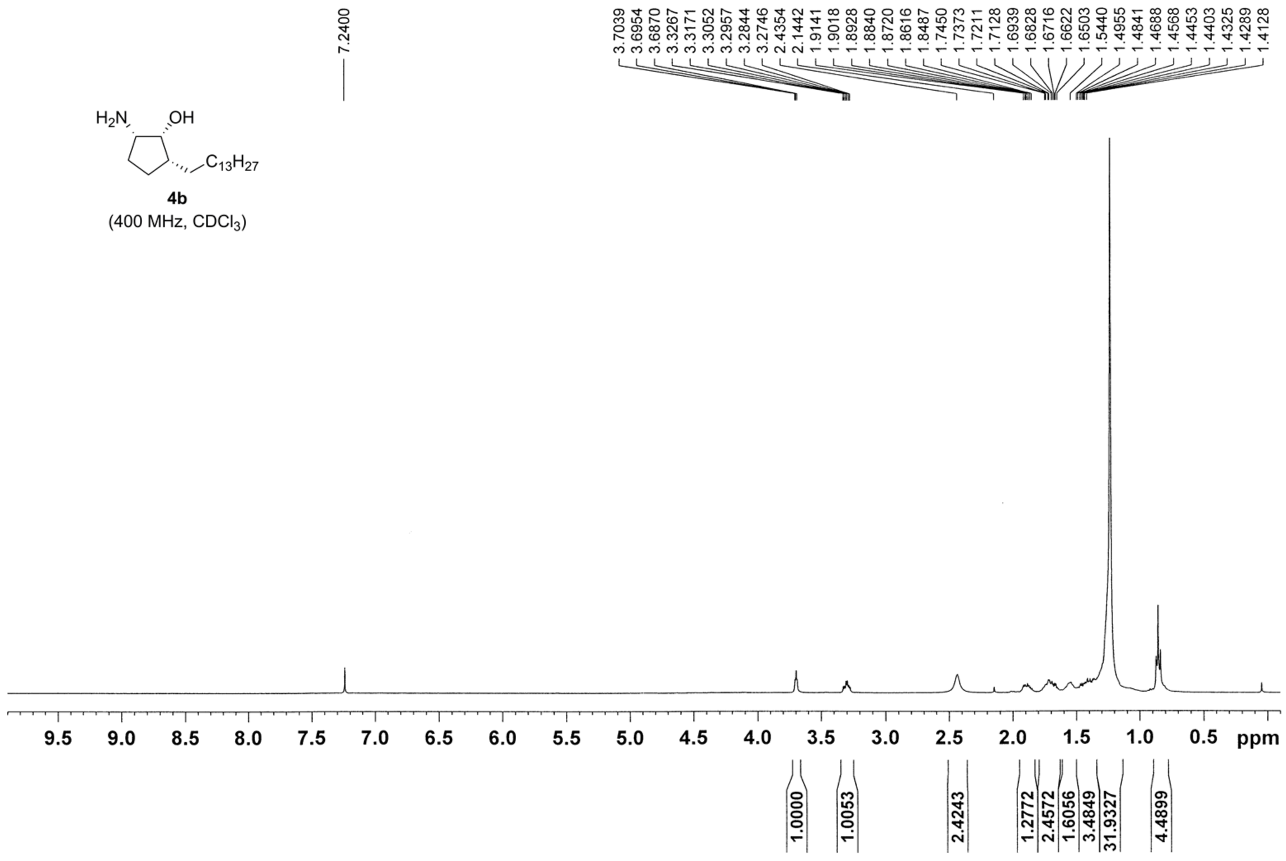

Figure S27. ^{1}H NMR spectrum of **4b**.

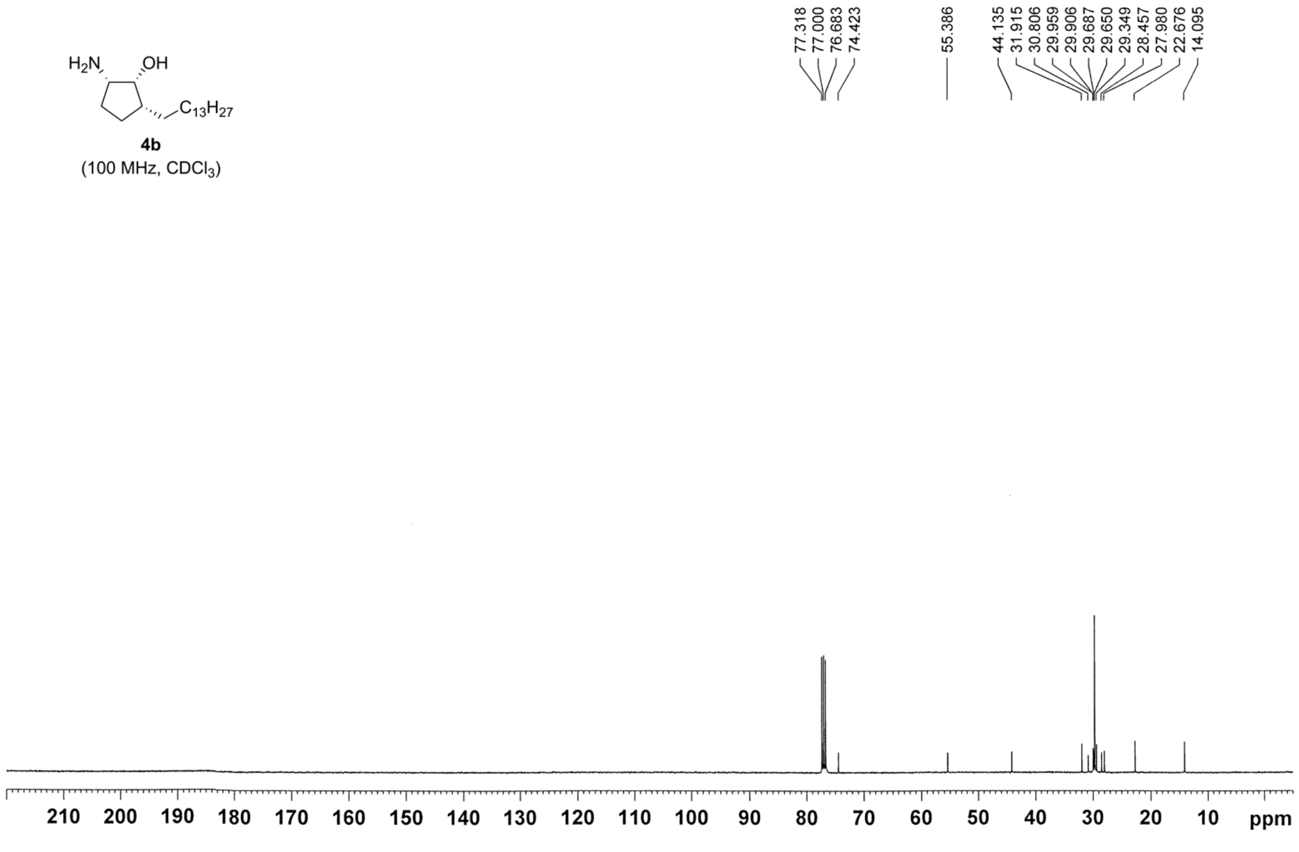

Figure S28. ^{13}C NMR spectrum of **4b**.

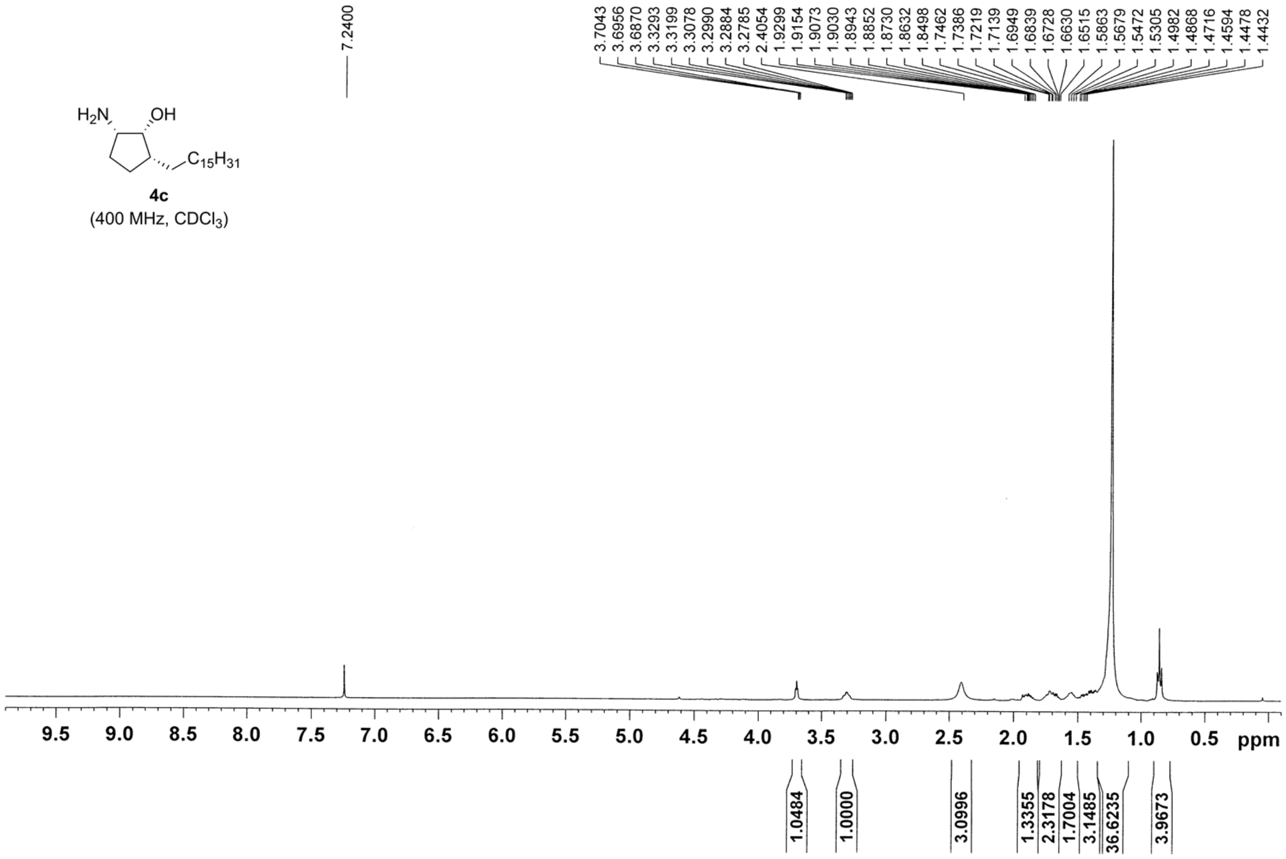

Figure S29. ^{1}H NMR spectrum of **4c**.

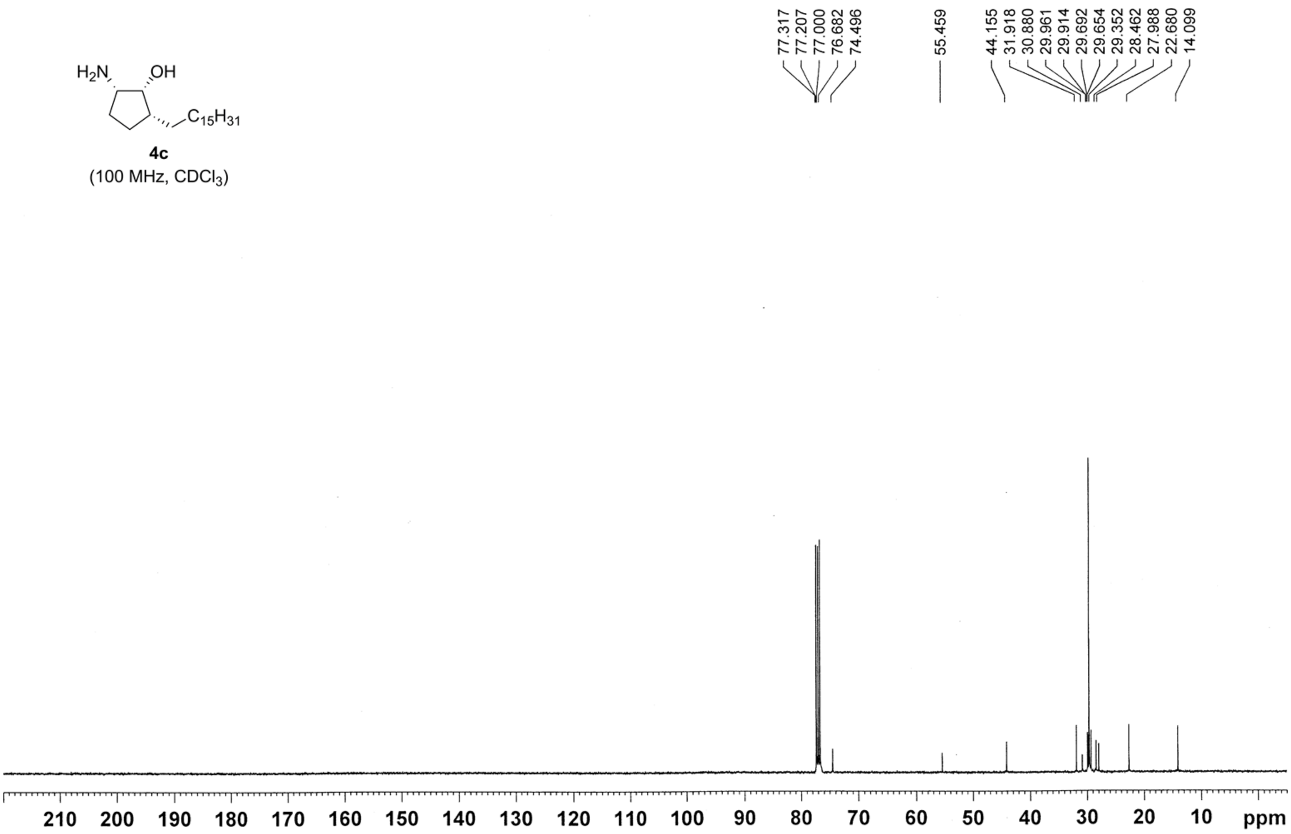

Figure S30. ^{13}C NMR spectrum of **4c**.

© 2015 by the authors; licensee MDPI, Basel, Switzerland. This article is an open access article distributed under the terms and conditions of the Creative Commons Attribution license (http://creativecommons.org/licenses/by/4.0/).
